# Accumulation of the RNA polymerase subunit RpoB depends on RNA editing by OsPPR16 and affects chloroplast development during early leaf development in rice

**DOI:** 10.1111/nph.16769

**Published:** 2020-07-22

**Authors:** Weifeng Huang, Yang Zhang, Liqiang Shen, Qian Fang, Qun Liu, Chenbo Gong, Chen Zhang, Yong Zhou, Cui Mao, Yongli Zhu, Jinghong Zhang, Hongping Chen, Yu Zhang, Yongjun Lin, Ralph Bock, Fei Zhou

**Affiliations:** ^1^ National Key Laboratory of Crop Genetic Improvement Huazhong Agricultural University Wuhan 430070 China; ^2^ Key Laboratory of Synthetic Biology CAS Center for Excellence in Molecular Plant Sciences Shanghai Institute of Plant Physiology and Ecology Chinese Academy of Sciences Shanghai 200032 China; ^3^ University of Chinese Academy of Sciences Beijing 100049 China; ^4^ College of Bioscience and Bioengineering Jiangxi Agricultural University Nanchan 330045 China; ^5^ Nanchang Subcenter of Rice National Engineering Laboratory Key Laboratory of Rice Physiology and Genetics of Jiangxi Province Rice Research Institute Jiangxi Academy of Agricultural Sciences Nanchang 330200 China; ^6^ Max‐Planck‐Institut für Molekulare Pflanzenphysiologie Am Mühlenberg 1 Potsdam‐Golm D‐14476 Germany

**Keywords:** Chl biosynthesis, chloroplast development, plastid, RNA editing, RNA polymerase, transcription

## Abstract

- Plastid‐encoded genes are coordinately transcribed by the nucleus‐encoded RNA polymerase (NEP) and the plastid‐encoded RNA polymerase (PEP). Resulting primary transcripts are frequently subject to RNA editing by cytidine‐to‐uridine conversions at specific sites. The physiological role of many editing events is largely unknown.
- Here, we have used the CRISPR/Cas9 technique in rice to knock out a member of the PLS‐DYW subfamily of pentatricopeptide repeat (PPR) proteins.
- We found that OsPPR16 is responsible for a single editing event at position 545 in the chloroplast *rpoB* messenger RNA (mRNA), resulting in an amino acid change from serine to leucine in the β‐subunit of the PEP. In striking contrast to loss‐of‐function mutations of the putative orthologue in *Arabidopsis*, which were reported to have no visible phenotype, knockout of *OsPPR16* leads to impaired accumulation of RpoB, reduced expression of PEP‐dependent genes, and a pale phenotype during early plant development. Thus, by editing the *rpoB* mRNA, *OsPPR16* is required for faithful plastid transcription, which in turn is required for Chl synthesis and efficient chloroplast development.
- Our results provide new insights into the interconnection of the finely tuned regulatory mechanisms that operate at the transcriptional and post‐transcriptional levels of plastid gene expression.

## Introduction

The chloroplast is the organelle where photosynthesis occurs, and its biogenesis and development are essential for plant development and crucial to crop productivity. Chloroplasts develop from proplastids, undifferentiated plastids that are present in meristems and embryonic tissues (Demarsy *et al*., 2006). In the absence of light, proplastids turn into etioplasts, a type of plastid that is arrested in chloroplast development. Upon illumination, proplastids and etioplasts develop into chloroplasts (Pogson *et al*., 2015; Armarego‐Marriott *et al*., 2019). The process of chloroplast development can be divided into three steps (Zhang *et al*., 2017). In the first step, plastid DNA synthesis and replication are activated. In the second step, genes encoding the plastid gene expression machinery are preferentially transcribed by nucleus‐encoded RNA polymerase (NEP) for rapid establishment of the plastid genetic system, leading to a strong increase in the transcription and translation levels of plastid‐encoded genes. In the final step, the activity of plastid‐encoded RNA polymerase (PEP) increases and is maintained at a high level, thus leading to strong expression of plastid‐encoded components of the photosynthetic apparatus. By contrast, NEP activity is reduced and kept at a low basal level. Impairments in the chloroplast biogenesis program result in a variety of abnormal phenotypes, such as embryo lethality, pigment‐deficient (e.g. albino) leaves, and retarded growth (Ruppel & Hangarter, 2007; Kadirjan‐Kalbach *et al*., 2012; Su *et al*., 2012; Tanoue *et al*., 2014; Lin *et al*., 2015; Lin *et al*., 2019; Wang *et al*., 2019; Xu *et al*., 2019). For example, in the *pgp1pgp2* double mutant of *Arabidopsis*, the biosynthesis of phosphatidylglycerol (a major thylakoid membrane phospholipid) is blocked. This leads to impaired thylakoid membrane biogenesis and inhibition of chloroplast development, which in turn compromises seed germination (Tanoue *et al*., 2014). In a rice (*Oryza sativa*) mutant, a single base change in *OsValRS2* (encoding an organellar Val‐tRNA synthetase) resulted in impaired chloroplast gene expression, in turn causing inefficient ribosome biogenesis, virescent to albino phenotypes in seedlings, and white panicles at heading (Wang *et al*., 2016). Although many genes involved directly or indirectly in chloroplast development have been identified, their complex interplay in the regulation of the biogenesis program is still far from being understood.

The chloroplast genome of seed plants contains approximately 130 genes, including 30 genes for transfer RNAs (tRNAs), approximately 30 genes related to plastid gene expression, and about 50 genes encoding photosynthesis‐associated proteins (Sugiura, 1992; Wakasugi *et al*., 2001; Kahlau *et al*., 2006; Daniell *et al*., 2016). The chloroplast has its own transcription and translation systems. Plastid‐encoded genes are coordinately transcribed by two RNA polymerases (RNAPs), dubbed NEP and PEP (Hedtke *et al*., 1997; Hess & Börner, 1999; Liere & Börner, 2007). NEP is encoded by *RpoTp* in the nuclear genome. PEP is composed of four core subunits (encoded by the plastid *rpoA*, *rpoB*, *rpoC1*, and *rpoC2* genes), sigma factors, and a set of polymerase‐associated proteins encoded by nuclear genes. Plastid genes can be divided into three classes, depending on their promoter structures (Hajdukiewicz *et al*., 1997): PEP‐dependent genes (class I), PEP and NEP‐dependent genes (class II), and NEP‐dependent genes (class III). Photosynthesis‐related genes and most genes for tRNAs are PEP‐dependent genes. The four genes encoding the core subunits of PEP (*rpoA*, *rpoB*, *rpoC1*, and *rpoC2*) and the *rpl23* gene (encoding ribosomal protein large subunit 23) were identified as NEP‐dependent genes (Zhelyazkova *et al*., 2012; Williams‐Carrier *et al*., 2014; Börner *et al*., 2015). When NEP or PEP functions are disturbed, the transcription of plastid genes becomes unbalanced, leading to abnormal chloroplast development and plant growth (Allison *et al*., 1996; Legen *et al*., 2002; Hricova *et al*., 2006; Courtois *et al*., 2007; Swiatecka‐Hagenbruch *et al*., 2008; Bock *et al*., 2014).

Pentatricopeptide repeat (PPR) proteins have been implicated in nearly all post‐transcriptional steps in plastid gene expression, including RNA editing (Cai *et al*., 2009; Toda *et al*., 2012; Hayes *et al*., 2015; Yap *et al*., 2015; Wang *et al*., 2017; Small *et al*., 2019), processing (Kazama & Toriyama, 2003; Hattori *et al*., 2007; Fujii *et al*., 2016; Wu *et al*., 2016), translation (Barkan *et al*., 2012; Zoschke *et al*., 2016), intron splicing (Hsieh *et al*., 2015; Aryamanesh *et al*., 2017; Ito *et al*., 2018; Sun *et al*., 2018; Wu *et al*., 2019), and messenger RNA (mRNA) stability (Beick *et al*., 2008; Hammani *et al*., 2016). In plastids of flowering plants, RNA editing results in alteration of the genetic information at the mRNA level through cytidine‐to‐uridine conversions at highly specific sites (Ichinose & Sugita, 2016; Lu, 2018). Several types of trans‐acting protein factors have been implicated in RNA editing (Chateigner‐Boutin *et al*., 2008; Tillich *et al*., 2009; Bentolila *et al*., 2012; Takenaka *et al*., 2012; Zhang *et al*., 2017; Sandoval *et al*., 2019; Small *et al*., 2019), with PPR proteins of the DYW type being the key factors that mediate the sequence‐specific recognition of editing sites (Okuda *et al*., 2009; Zhou *et al*., 2009; Barkan & Small, 2014; Jiang *et al*., 2018; Sun *et al*., 2018). A total of 491 PPR proteins have been found in rice (Chen *et al*., 2018), and 131 of these contain a DYW domain, suggesting that they could act as RNA editing factors (Salone *et al*., 2007). Of the rice DYW‐PPR proteins, 30 are predicted to be chloroplast localized. So far, 24 RNA editing sites have been identified in the rice plastid genome (Corneille *et al*., 2000), but which PPR proteins recognize these sites is not well understood. To date, only five rice PPR proteins (WSL5, DUA1, OsPPR4, OsPPR6 and OsPGL1) have been assigned to specific plastid RNA editing sites and implicated in the editing of altogether seven sites (Asano *et al*., 2013; Tang *et al*., 2017; Cui *et al*., 2018; Liu *et al*., 2018; Xiao *et al*., 2018). Also, although transplastomic studies in tobacco (Bock *et al*., 1994; Karcher *et al*., 2008; Loiacono *et al*., 2019) and mutant analyses in *Arabidopsis* (Robbins *et al*., 2009; Zhou *et al*., 2009; Bentolila *et al*., 2012; Ramos‐Vega *et al*., 2015) have revealed the importance of some RNA editing events in chloroplasts, the functional significance of many others remains unknown.

To investigate the effects of RNA editing on chloroplast development in rice, we have begun to use the CRISPR/Cas9 technique to knock out the 30 chloroplast‐localized PPR proteins containing a DYW motif. Here, we describe the generation and characterization of such knockout mutants for the *OsPPR16* gene. We show that targeted inactivation of this DYW‐PPR gene results in a striking developmental phenotype, with leaves being pale during early plant development, whereas later leaves are largely unaffected. Analysis of all chloroplast editing sites revealed that editing of a single site in *rpoB* was defective in *osppr16* mutants, resulting in decreased accumulation of the RpoB protein and low PEP activity. We provide evidence for low transcription of the *trnE* gene, resulting in strongly decreased Chl synthesis that in turn delays early chloroplast development.

## Materials and Methods

### Plant material and growth conditions

Rice cv Zhonghua 11 (*Oryza sativa* L. ssp. *geng/japonica* cv Zhonghua 11) was used in this study. *OsPPR16* transgenic plants and Zhonghua 11 (as the wild‐type, WT) were planted in the experimental field of Huazhong Agricultural University, Wuhan, China (30.4°N, 114.2°E).

To analyze the phenotype of *OsPPR16* loss of function, T_1_ plants were used. To this end, seeds were germinated on half‐strength MS medium (Murashige & Skoog, 1962) in a tissue culture room at 28°C under a 14 h : 10 h, light : dark, photoperiod until the five‐leaf stage. The seedlings were then transferred to the field and grown to maturity.

For norflurazon (NF; Sigma Aldrich) treatments, NF was added to the culture medium at 5 μM final concentration. Seeds were surface sterilized and then cultured with or without NF at 28°C under continuous illumination.

### Knockout and knockdown of *OsPPR16*

The CRISPR/Cas9 technology was used to knock out *OsPPR16*. Two CRISPR/Cas9 vectors were constructed, as previously described (Ma *et al*., 2015). Three specific target sequences for *OsPPR16* were designed using CRISPR‐P (Lei *et al*., 2014). Target1 and Target2 were cloned into the destination vector pYLCRISPR/Cas9‐MH, generating plasmid pYLCRISPR‐osppr16d, and Target3 was cloned into the destination vector pYLCRISPR/Cas9‐MH, generating pYLCRISPR‐osppr16s. The CRISPR vectors were introduced into rice cv Zhonghua 11 by *Agrobacterium*‐mediated transformation, as previously described (Hiei *et al*., 1994). The genotype of CRISPR/Cas9 plants was analyzed by PCR and direct sequencing of the amplification products. The sequences of targets and PCR primers are listed in Supporting Information Table S1.

RNA interference (RNAi) by double‐stranded RNA expression was used to knock down *OsPPR16*. An RNAi vector was constructed by amplifying a 275 bp fragment using Zhonghua 11 complementary DNA (cDNA) as a template and primers RNAi16‐F and RNAi16‐R (Table S1). The resulting amplification product was inserted into vector pDS1301 (Dai *et al*., 2007). The recombinant binary vector was transformed into Zhonghua 11 callus material. The relative expression of *OsPPR16* in transgenic RNAi plants was analyzed by quantitative real‐time PCR (qRT‐PCR) using Real16‐F and Real16‐R as primers (Table S1).

### Transmission electron microscopy

WT and *osppr16d* mutant leaves at the three and five‐leaf stages of plants were collected for transmission electron microscopy (TEM) processing as previously described (Leng *et al*., 2017).

### RNA isolation and Northern blot analysis

Total RNA was extracted from leaf tissue using the Trizol reagent and following the manufacturer’s protocol (Invitrogen, Carlsbad, CA, USA). Northern blot analysis was performed as previously described (Kazama *et al*., 2008). Hybridization probes were labeled with digoxigenin (DIG) by PCR adding 0.01 μM DIG‐deoxyuridine triphosphate to the reaction mixture. The sequences of all primers are listed in Table S1.

### Measurement of Chl contents

Samples of 0.015–0.035 g of fresh leaves were extracted with 1.8 ml of Chl extraction solution (volume ratio of ethanol : acetone : water 4.5 : 4.5 : 1). Chl content was measured using published methods (Mao *et al*., 2011).

### Subcellular localization analyses

The coding region of *OsPPR16* with the exception of the stop codon was amplified using genomic DNA as template and cloned into the pXCSG‐YFP vector (Feys *et al*., 2005). Rice protoplasts were transformed as described previously (Zhang *et al*., 2011). The yellow fluorescent protein (YFP) and the Chl fluorescence signal of the chloroplast were observed and photographed using a confocal microscope (FV 1200; Olympus, Toyko, Japan) after 16 h of dark culture of the transfected protoplasts at 28°C.

To further confirm the localization of OsPPR16 within the chloroplast, the coding region of *OsPPR16* (without the stop codon) was cloned into the pAN580‐GFP vector (Yang *et al*., 2018) to generate pAN580‐OsPPR16GFP and co‐transformed with a PEND‐CFP construct (which expresses a fluorescent marker protein for plastid nucleoids; Terasawa & Sato, 2005) into rice protoplasts. The YFP, cyan fluorescent protein (CFP), and the Chl fluorescence signal of the chloroplast were subsequently observed by confocal laser‐scanning microscopy.

### Sequence analyses and phylogenetic studies

The coding sequences (CDSs) of *rpoB*, *ndhB*, and *ndhD* in 79 land plants, whose plastid and nuclear genomes had been sequenced and annotated, were downloaded from the database of the National Center for Biotechnology Information (NCBI, https://www.ncbi.nlm.nih.gov). Homologous sequences of OsPPR16 in 79 land plants were identified using the NCBI’s Blastp search program. Multiple sequence alignments were conducted by Geneious 4.8.3 (www.geneious.com). The nucleotides corresponding to the editing sites of *rpoB*‐545, *ndhB*‐746, and *ndhD*‐887 were determined based on the aligned CDSs. A phylogenetic tree was constructed using Mega 5.0 (Tamura *et al*., 2011) and the neighbor‐joining method with 1000 bootstrap replicates. The accession numbers of RpoB, NdhB, NdhD, and the homologous of OsPPR16 are listed in Table S2.

### Analysis of RNA editing, splicing, and relative gene expression

Total RNA was extracted with the Transzol reagent (TransGen Biotech Co. Ltd, Beijing, China). Thereafter, 2 μg of total RNA was reverse transcribed using M‐MLV reverse transcriptase (Invitrogen) and random primers to obtain cDNA according to the manufacturer’s instructions. Fragments containing editing sites were amplified by reverse transcription (RT)‐PCR using cDNA as template and previously described methods (Zhang *et al*., 2017). The RT‐PCR products were sequenced directly using the chain termination method. The sequences of all primers are listed in Table S1. RNA splicing was analyzed as described previously (Tan *et al*., 2014) using specific primers flanking the introns (Table S1). Relative gene expression was analyzed by qRT‐PCR using a FastStart Universal SYBR Green Master (Roche, Basel, Switzerland) on an ABI Wuantstudio 6 Flex Real‐Time PCR system. The amplification program was as follows: 95°C for 10 min, followed by 40 cycles of 95°C for 15 s and 60°C for 1 min. Relative gene expression was analyzed using the $2^{‐\Delta \Delta C_{T}}$ method (Livak & Schmittgen, 2001). Rice triosephosphate isomerase (*OsTPI*) was used as reference gene (Wang *et al*., 2016). Primer sequences are listed in Table S1.

### Western blot analyses

Total protein samples of *osppr16d* and WT at the three and five‐leaf stages were isolated with an extraction buffer containing 50 mM Tris hydrochloride (pH 8.0), 150 mM sodium chloride, 1% NP40, and 2× protease inhibitor (COMPLETE; Roche). Protein concentrations were determined according to a previously described protocol (Bradford, 1976). Proteins were separated by sodium dodecyl sulfate polyacrylamide gel electrophoresis, semi‐dry transferred onto polyvinylidene difluoride membranes, and incubated with antibodies. Signals were detected using the Clarity Western ECL Substrate kit (Bio‐Rad, Hercules, CA, USA) and visualized with an imaging system (ChemiDocTMXRS; Bio‐Rad). Heat‐shock protein 82 (HSP82) was used as an internal control. Antibodies against RpoB and HSP82 were obtained from Phytoab (www.phytoab.com) and BGI (Shenzhen, China), respectively. Antibodies against AtpB, PetB and PsaB were purchased from Agrisera (www.agrisera.com).

## Results

### Identification and characterization of the *osppr16* mutant

**Fig. 1:**
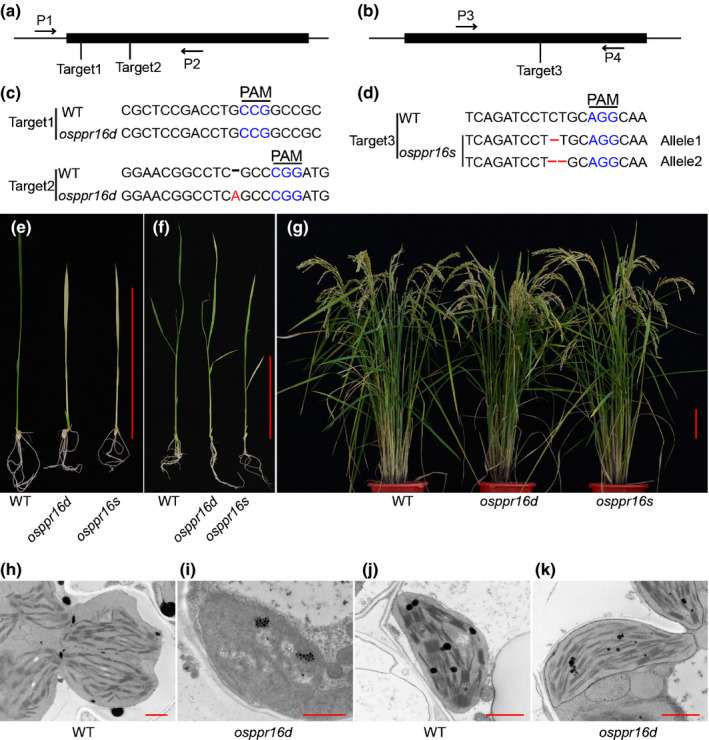
Generation and characterization of rice (*Oryza sativa*) *osppr16* mutants. (a) Strategy I and (b) Strategy II for CRISPR/Cas9‐dependent gene editing of *OsPPR16*. Mutants obtained with Strategy I and Strategy II and selected for in‐depth characterization were named *osppr16d* and *osppr16s*, respectively. (c) Sequences around the two CRISPR targets (Target1 and Target2) in wild‐type (WT) and *osppr16d*. PAM, protospacer adjacent motif. (d) Sequence around Target3 in WT and *osppr16s*. (e) *osppr16d*, *osppr16s*, and WT plants at the three‐leaf stage. Bar, 10 cm. (f) *osppr16d*, *osppr16s* and WT plants at the five‐leaf stage. Bar, 10 cm. (g) *osppr16d*, *osppr16s*, and WT plants at seed set. Bar, 10 cm. (h, i) Chloroplast ultrastructure of (h) WT and (i) *osppr16d* at the three‐leaf stage. Bars, 1 μm. (j, k) Chloroplast ultrastructure of (j) WT and (k) *osppr16d* plants at the five‐leaf stage. Bars, 1 μm.

To investigate the functional role of RNA editing in chloroplast biology in rice, we have begun to use the CRISPR/Cas9 technique to systematically knock out all members of the PLS‐DYW subfamily of PPR proteins, which are predicted to localize to the chloroplast. A striking developmental phenotype was observed in leaves of *osppr16* mutants, which were pale at the early developmental stage, but normally green at later stages (Fig. 1). This interesting developmental gradient prompted us to investigate the function of *OsPPR16* in detail.

Two strategies were applied to knock out *OsPPR16*. One strategy involved a vector (pYLCRISPR‐osppr16d) that expresses two guide RNAs targeting two sites in the *OsPPR16* gene (Fig. 1a). Thirty‐six T_0_ transgenic plants were obtained, 11 of which showed pale early leaves of tillers that gradually turned green (Fig. S1). At later developmental stages, no significant difference in leaf color was observed between transgenic and WT plants (Figs 1, S1). The other 25 transgenic lines were phenotypically indistinguishable from WT plants during the entire growth period. The genotype of the 36 T_0_ transgenic plants was assayed by direct sequencing of PCR products generated by amplification of the *OsPPR16* locus (Fig. 1a). These analyses revealed that the gene was edited at Target1 and/or Target2 in the 11 lines with pale early leaves in tillers, whereas the 25 WT‐like lines had the WT *OsPPR16* sequence or were heterozygous (Table S3).

Another CRISPR/Cas9 vector (pYLCRISPR‐osppr16s) was constructed that expresses a single guide RNA (Strategy II) that targets a site in the 3′ half of the *OsPPR16* gene (Fig. 1b) and transformed into rice callus cells. This independent strategy was chosen first to provide additional evidence for the mutant phenotype being causally related to inactivation of the *OsPPR16* gene, and second to rule out the possibility of off‐target genome editing by CRISPR/Cas9 (Fu *et al*., 2013; Lin *et al*., 2014; Jin *et al*., 2019). Thirty‐four T_0_ transgenic plants were obtained, 10 of which had the same phenotype as the mutant lines generated by Strategy I (Fig. S1c). The remaining 24 lines had a WT‐like phenotype, and their genotyping revealed that they were unmutated in *OsPPR16* or heterozygous (Table S3). Taken together, these results strongly suggest that inactivation of *OsPPR16* leads to pale early leaves of tillers in rice.

Two independently generated *osppr16* lines were selected for further characterization: one line from Strategy I (named *osppr16d*) and one line from Strategy II (named *osppr16s*). In the *osppr16d* mutant, no base change was detected at Target1, whereas an additional adenine was inserted at Target2, resulting in a frameshift mutation (Fig. 1c). *osppr16s* represents a biallelic mutant, in which one nucleotide (C) was deleted in allele 1 and two nucleotides (CT) were deleted in allele 2 at Target3 (Fig. 1d). The leaves of both *osppr16d* and *osppr16s* T_1_ seedlings were pale during early leaf development (Fig. 1e) and turned green during the five‐leaf stage (Fig. 1f). A very similar phenotype of T_1_ plants was seen for T_0_ plants when they were transferred to the paddy field (Fig. S1). By contrast, leaf color of adult plants grown in the glasshouse or in the field was not different from the WT (Figs 1g, S1).

To investigate the effect of *osppr16* mutation on chloroplasts biogenesis, chloroplast ultrastructure at the three‐leaf and five‐leaf stages was analyzed by TEM. Whereas chloroplasts in WT leaves showed well‐structured thylakoids at both the three‐leaf (Fig. 1h) and five‐leaf stages (Fig. 1j), the chloroplasts of *osppr16d* leaves were small and lacked organized thylakoid membranes at the three‐leaf stage (Fig. 1i). By contrast, chloroplasts containing well‐organized thylakoids (and stacked grana) were present at the five‐leaf stage (Fig. 1k). These results indicate that the OsPPR16 gene product plays an important role in the biogenesis of chloroplasts during early leaf development.

### Chl synthesis in *osppr16* is reduced during early leaf development

**Fig. 2:**
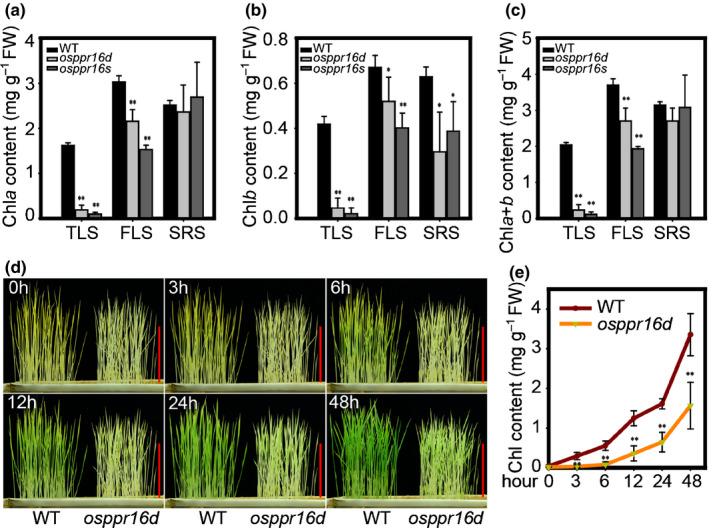
Analysis of Chl synthesis in rice (*Oryza sativa*) wild‐type (WT) and *osppr16* mutant plants. (a–c) Chl content of leaves at different development stages in *osppr16* and WT plants. The contents of (a) Chl*a*, (b) Chl*b*, and (c) the total Chl are shown. TLS, three‐leaf stage; FLS, five‐leaf stage; SRS, seed ripening stage. Error bars indicate SD (*n* = 4), *, *P* < 0.05; **, *P* < 0.01 (Student’s *t*‐test). (d) Time course of de‐etiolation of WT and *osppr16* seedlings. Plants were photographed 0, 3, 6, 12, 24, and 48 h after light treatment. Bars, 10 cm. (e) Chl contents in leaves of WT and *osppr16d* during the de‐etiolation time course. Error bars indicate SD (*n* = 4), **, *P* < 0.01 (Student’s *t*‐test).

To further characterize the pigment‐deficient phenotype of the *osppr16* mutants, the Chl content of leaves at different stages was analyzed (Fig. 2). The Chl*a* contents in *osppr16d* and *osppr16s* leaves were 14.2% and 5.9%, respectively, of that in the WT at the three‐leaf stage and 71.1% and 70.9%, respectively, of the WT level at the five‐leaf stage. By contrast, no significant difference in Chl*a* content was detectable between *osppr16* and the WT at the time of seed set (Fig. 2a). The Chl*b* contents in *osppr16d* and *osppr16s* leaves were also much lower than in the WT at the three‐leaf stage, and, similar to Chl*a* contents, partially recovered at the five‐leaf stage. However, in contrast to Chl*a* contents, no full recovery to WT levels was seen in adult plants (Fig. 2b), although their total Chl content was not significantly different from that of the WT (Fig. 2c).

To further investigate the effect of *OsPPR16* on Chl synthesis, seeds of WT and *osppr16d* were germinated in soil in the dark at 28°C for 8 d and then treated with light. The Chl content of WT and *osppr16d* seedlings was analyzed at 0, 3, 6, 12, 24 and 48 h after lighting. After 3 h of lighting, the WT seedlings slightly turned green and the green color became obvious after 6 h of light treatment. By contrast, the greening of *osppr16d* seedlings was considerably delayed, and the seedlings did not turn visibly green until 12 h after lighting (Fig. 2d). After 48 h of de‐etiolation treatment, mutant seedlings were still only light green, whereas WT seedlings had virtually fully greened (Fig. 2d). The time course of Chl accumulation corresponded well with the visual observation of the greening process (Fig. 2e). These results suggest that inactivation of *OsPPR16* reduces the rate of Chl synthesis or Chl stability.

### OsPPR16 is a chloroplast‐localized PLS‐DYW subfamily PPR protein

**Fig. 3:**
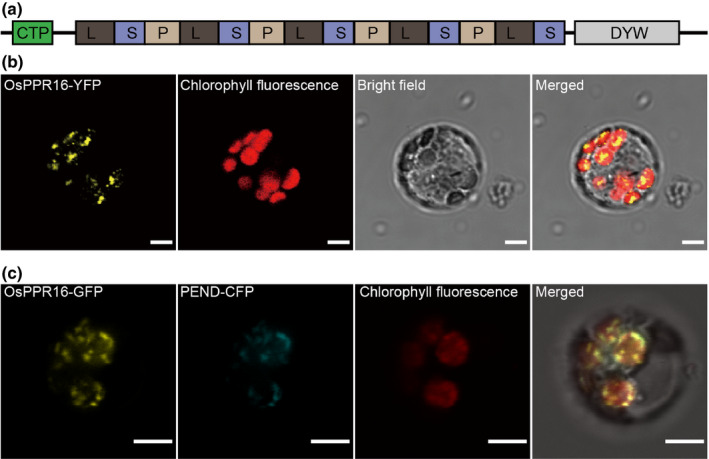
Domain structure and subcellular localization analysis of OsPPR16. (a) Structure of OsPPR16. CTP, chloroplast transit peptide. (b) Subcellular location of OsPPR16 in rice protoplasts. YFP, yellow fluorescent protein. Bars, 5 µm. (c) Co‐localization of OsPPR16‐GFP (yellow) and PEND‐CFP (cyan) in chloroplast nucleoids. Note that the GFP fluorescence was detected in the YFP channel, and therefore is represented as yellow color. GFP, green fluorescent protein; CFP, cyan fluorescent protein. Bars, 5 µm.

Sequence analysis showed that the *OsPPR16* gene contains a single exon (Fig. 1a). *OsPPR16* is predicted to encode a PPR protein of 734 amino acids. Bioinformatics analysis of the sequence revealed that OsPPR16 consists of 14 PPR motifs, a typical DYW motif (representing the putative cytidine deaminase; Oldenkott *et al*., 2019), and a chloroplast transit peptide (Fig. 3a). Homologous proteins were identified by NCBI’s Blast to analyze the relationship between OsPPR16 and similar proteins in other species. The searches revealed that OsPPR16 shares 57.7% amino acid sequence identity with the PPR PLS‐DYW subfamily protein CRR22 in *Arabidopsis thaliana* (Okuda *et al*., 2009; Fig. S2). Phylogenetic tree analysis showed that proteins homologous to OsPPR16 are widely distributed across land plants (Fig. S3). CRR22 (encoded by At1g11290) was shown to be essential for the editing of the *ndhB*‐746, *ndhD*‐887 and *rpoB*‐551 sites in *Arabidopsis* (Okuda *et al*., 2009). The similarity of OsPPR16 to CRR22 suggested that OsPPR16 is a typical PLS‐DYW PPR protein and may also be involved in organellar RNA editing.

OsPPR16 was predicted to localize to the chloroplast by ChloroP (www.cbs.dtu.dk/services/ChloroP/) and Plant‐mPLoc (http://www.csbio.sjtu.edu.cn/bioinf/plant‐multi/). To experimentally verify this prediction, a transformation vector expressing an OsPPR16‐YFP fusion protein was constructed and introduced into rice protoplasts. Analysis of transformed protoplasts by confocal laser‐scanning microscopy showed that the yellow fluorescence of OsPPR16‐YFP exclusively co‐localized with the Chl fluorescence of the chloroplasts (Fig. 3b). Interestingly, the YFP fluorescence was concentrated in small dot‐like structures within the transfected chloroplasts. Many chloroplast RNA‐binding proteins are associated with nucleoids, presumably because RNA processing initiates co‐transcriptionally (Majeran *et al*, 2012). To examine whether the dot‐like structures represent nucleoids, OsPPR16‐GFP was co‐expressed with PEND‐CFP, a fluorescent marker protein for plastid nucleoids (Terasawa & Sato, 2005). Indeed, the green fluorescent protein signals co‐localized with the CFP signals, indicating that OsPPR16 associates with chloroplast nucleoids (Fig. 3c).

### Editing of *rpoB*‐545 is impaired in *osppr16* mutant plants

**Fig. 4:**
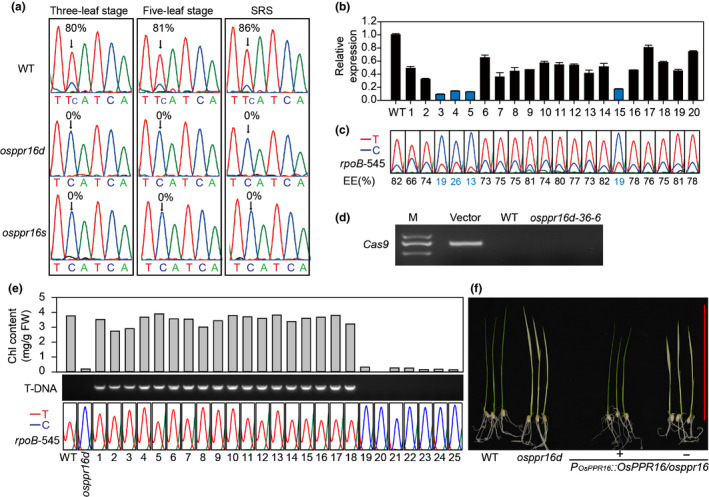
Editing analysis of *rpoB*‐545 in rice (*Oryza sativa*) wild‐type (WT), *osppr16*, RNA interference (RNAi), and complemented plants. (a) Editing efficiency of *rpoB*‐545 in WT and *osppr16* leaves at three‐leaf, five‐leaf, and seed ripening stages. SRS, seed ripening stage. The arrows refer to the editing site. (b) Relative expression of *OsPPR16* in RNAi transgenic plants, as determined by quantitative real‐time PCR. *OsTPI* was used as reference gene. (c) Editing efficiency of *rpoB*‐545 in *OsPPR16* RNAi lines. EE, editing efficiency. (d) Assay for the *Cas9* gene by PCR in *osppr16d* T_1_ plants. M, marker. Vector, pYLCRISPR‐osppr16d. The *osppr16d‐36‐6* plant was confirmed as *Cas9*‐free line, and subsequently used to conduct complementation analysis. (e) Analysis of Chl content, transfer DNA (T‐DNA) presence, and RNA editing efficiency in WT, *osppr16d*, and T_1_ seedlings from a T_0_ complemented line at the three‐leaf stage. (f) Phenotypes of WT, *osppr16d*, and complemented T_1_ seedlings (*P_OsPPR16_::OsPPR16*/*osppr16*). +, positive T_1_ seedlings segregated from a T_0_ complementation line; −, negative T_1_ seedlings segregated from a T_0_ complementation line. Bar, 10 cm.

Many PPR proteins, especially members of the PLS‐DYW subfamily, are involved in organellar RNA editing (Salone *et al*., 2007; Sun *et al*., 2016). To test whether this was the case for OsPPR16, the editing efficiency of all 24 editing sites in the rice chloroplast genome was examined in WT and *osppr16d* mutant leaves. cDNA sequencing revealed that editing of *rpoB*‐545 in *osppr16d* mutant plants was undetectable, whereas the other two editing sites in the *rpoB* mRNA (*rpoB*‐467 and *rpoB*‐560) and all other 21 editing sites in rice chloroplasts were edited normally and with similar efficiency as in the WT (Figs 4a, S4). RT‐PCR analysis also showed that splicing of all plastid introns occurred with similar efficiency as in the WT (Fig. S5). These results indicate that OsPPR16 is an editing factor specific for *rpoB*‐545.

RNAi was used to knock down *OsPPR16* and determine the effect of reduced OsPPR16 expression on *rpoB*‐545 editing. Twenty T_0_ transgenic plants were generated, and the expression of *OsPPR16* in lines 3, 4, 5 and 15 was determined to be less than 20% of that in the WT (Fig. 4b). When plastid RNA editing was assayed, the editing efficiency of *rpoB*‐545 in these RNAi lines was found to be more than 74% lower than that in the WT (Fig. 4c). In the other 16 lines, the expression level of *OsPPR16* was only mildly decreased, correlating with an only slightly reduced editing efficiency of *rpoB*‐545 (Fig. 4b,c). These results confirm that OsPPR16 is required for the editing of *rpoB*‐545 in rice plastids.

Genetic complementation studies were also performed (according to Methods S1) to ultimately confirm that OsPPR16 was responsible for the mutant phenotype of *osppr16*. To this end, *OsPPR16* fused to its native promoter was transformed into the *osppr16d‐36‐6* mutant line, which was free of the CRISPR/Cas9 construct (Fig. 4d). Chl contents, presence of the transfer DNA, and RNA editing efficiency were analyzed in 25 T_1_ seedlings from a T_0_ complemented line. The Chl content and editing efficiency of *rpoB*‐545 were similar to that of the WT in all seedlings that tested positive for the complementation construct and had green leaves at the three‐leaf stage (Fig. 4e,f). Similar to the *osppr16* mutant, seedlings not containing the complementation construct were Chl deficient and displayed impaired editing of *rpoB*‐545 (Fig. 4e,f). These results confirmed that OsPPR16 is required for the editing of *rpoB*‐545, and OsPPR16 disruption is the direct cause of the pale leaves of *osppr16* during early leaf development.

### Accumulation of the RpoB subunit of PEP is reduced in *osppr16* mutant plants

**Fig. 5:**
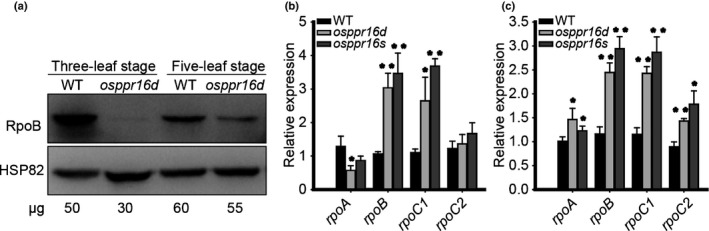
Protein and messenger RNA accumulation of plastid‐encoded RNA polymerase (PEP) core subunits in rice (*Oryza sativa*) wild‐type (WT) and *osppr16* mutant plants. (a) Immunoblot analysis of RpoB in WT and *osppr16* leaves at the three‐leaf and five‐leaf stages. An antibody against the heat‐shock protein HSP82 was used as an internal control. (b, c) Relative expression levels of the four genes encoding PEP core subunits in WT and *osppr16* leaves at (b) the three‐leaf stage and (c) the five‐leaf stage, as determined by quantitative real‐time PCR. *OsTPI* was used as reference gene. Error bars indicate SD (*n* = 3); *, *P* < 0.05; **, *P* < 0.01 (Student’s *t*‐test).

To investigate the effect of impaired editing of *rpoB*‐545 on the RpoB protein, the catalytic subunit of the PEP, the accumulation of RpoB was determined by Western blot analysis in *osppr16* and WT plants. At the three‐leaf stage, RpoB accumulation was much reduced in *osppr16* compared with the WT (Fig. 5a). At the five‐leaf stage, the accumulation of RpoB was partly restored in *osppr16* and only slightly lower than in WT plants at the same stage (Fig. 5a). Interestingly, accumulation of RpoB in the WT was considerably higher at the three‐leaf stage than at the five‐leaf stage (Fig. 5a). These results indicate that RpoB accumulation at an early developmental stage is severely affected in *osppr16*, presumably as a consequence of defective *rpoB*‐545 editing. In addition, the RpoB accumulation pattern in the WT suggests that more PEP is required for early chloroplast development than at later developmental stages.

PEP contains four core subunits encoded by the plastid genes *rpoA*, *rpoB*, *rpoC1* and *rpoC2*. The expression levels of *rpoA*, *rpoB*, *rpoC1* and *rpoC2* were analyzed by qRT‐PCR. At the three‐leaf stage, expression of *rpoB* and *rpoC1* in *osppr16* was strongly upregulated compared with the WT (Fig. 5b). At the five‐leaf stage, expression of *rpoB*, *rpoC1* and *rpoC2* was increased in *osppr16*, whereas *rpoA* was only slightly upregulated compared with the WT (Fig. 5c). These results suggest that the mRNA levels of *rpoB*, *rpoC1* and *rpoC2* (which are NEP‐dependent genes) respond to the low PEP activity and may be regulated by a feedback mechanism that likely involves the NEP.

### Structural basis of the PEP deficiency in the absence of editing at *rpoB*‐545

Impaired RNA editing of *rpoB*‐545 leads to reduced accumulation of the β‐subunit of PEP (Fig. 5a). To explore the possible molecular cause of the PEP deficiency in *osppr16* mutant plants, we analyzed the structural consequences of the lack of *rpoB* RNA editing at the protein level.

**Fig. 6:**
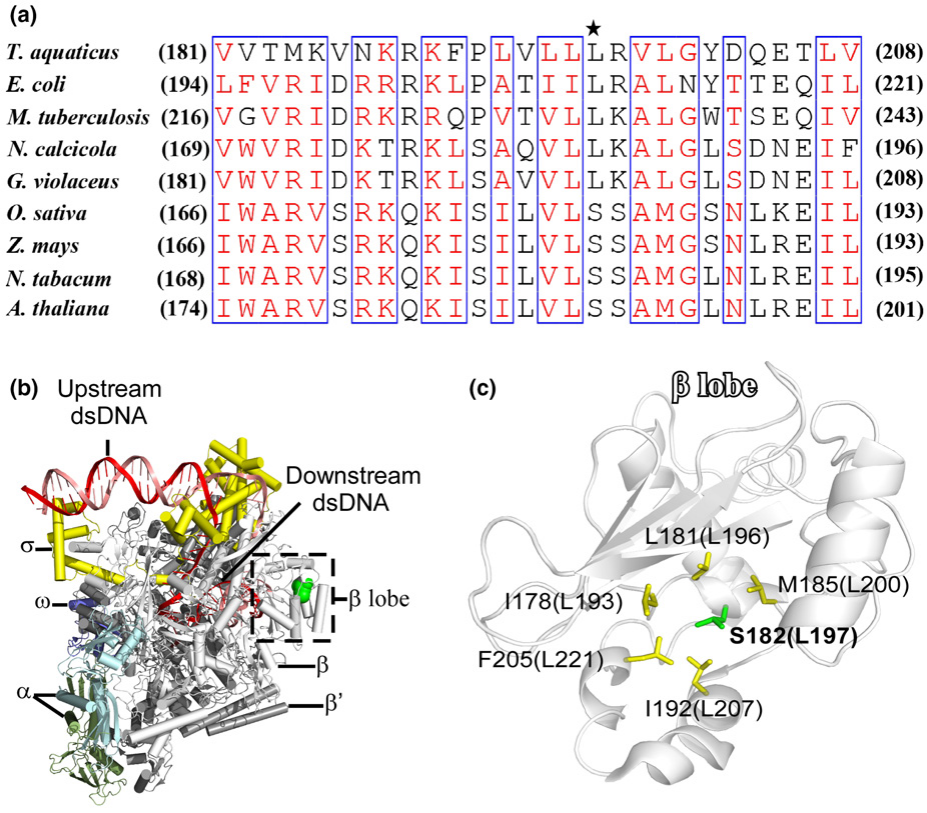
RNA editing at *rpoB*‐545 likely facilitates proper protein folding of the plastid‐encoded RNA polymerase (RNAP) β‐subunit. (a) Partial amino acid sequence alignment of RNAP β‐subunits of five bacterial species reveals a conserved leucine at the position corresponding to the edited codon (codon 182) of the rice chloroplast β‐subunit. By contrast, sequence alignment of chloroplast β‐subunits of four plant species reveals a serine at these positions (denoted by the asterisk). *T*.* aquaticus*,* Thermus aquaticus*; *E*.* coli*,* Escherichia coli*; *M*.* tuberculosis*,* Mycobacterium tuberculosis*; *N*.* calcicola*,* Nostoc calcicola*; *G*.* violaceus*,* Gloeobacter violaceus*; *O*.* sativa*,* Oryza sativa*; *Z*.* mays*,* Zea mays*; *N*.* tabacum*,* Nicotiana tabacum*; *A*.* thaliana*,* Arabidopsis thaliana*. (b) The crystal structure of *T*.* aquaticus* RNAP‐promoter open complex shows that L197 is located within the β‐lobe domain (dashed box) of the RNAP β‐subunit. The RNAP α, β, β′ and ω subunits are shown in cyan, gray, dark gray and blue, respectively, the promoter DNA is shown in red, and the σ‐factor is shown in yellow. (c) Amino acid L197 is buried inside the hydrophobic core of the β‐lobe domain. Numbers of amino acid residues correspond to the positions in the *T*.* aquaticus* RpoB protein. The equivalent positions in the *O*.* sativa* protein are given in parentheses.

Alignment of the amino acid sequences of the RNAP β‐subunits from five bacterial species (representing different phyla) shows a conserved leucine residue at the position corresponding to codon 182 of the rice *rpoB* gene (Fig. 6a). The crystal structure of the *Thermus aquaticus* RNAP‐promoter open complex shows that the conserved leucine (Leu^197^ in *T*.* aquaticus*) is buried inside the β‐lobe domain of the RNAP β‐subunit and is surrounded by hydrophobic residues (Fig. 6b,c; Bae *et al*., 2015), suggesting its importance in maintaining the hydrophobic core of the β‐lobe domain. In many plant species, the chloroplast gene for the β‐subunit of PEP contains a codon for a hydrophilic serine residue in the corresponding position (Fig. 6a), which is converted into a leucine codon by mRNA editing. Presence of a hydrophilic residue inside a hydrophobic domain potentially disrupts proper domain folding, thus decreasing protein stability and triggering proteolytic degradation (Dill & MacCallum, 2012). This explanation would be consistent with the reduced accumulation of the PEP β‐subunit in *osppr16* mutant plants (Fig. 5a) and underscores the functional importance of RNA editing to restore the conserved leucine residue.

### Decreased PEP activity in *osppr16* mutant plants during early leaf development

**Fig. 7:**
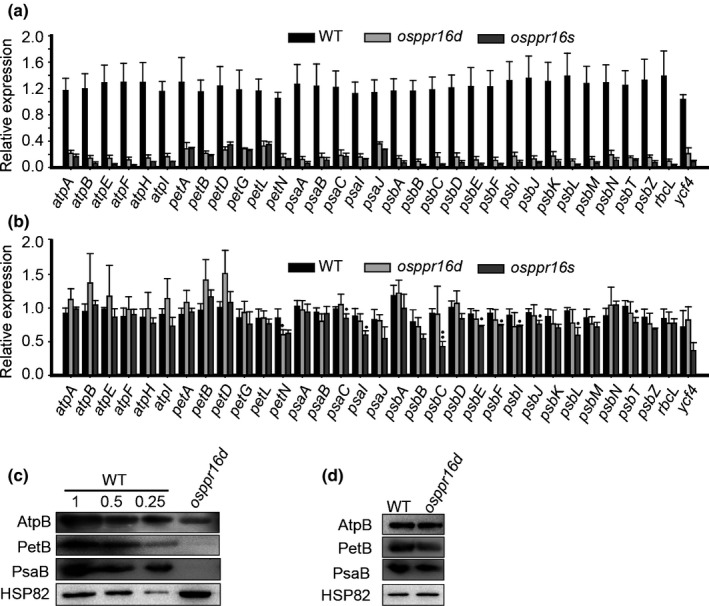
Transcript accumulation of plastid‐encoded RNA polymerase (PEP)‐dependent genes in rice (*Oryza sativa*) wild‐type (WT) and *osppr16* mutant leaves. (a, b) Quantitative real‐time PCR analysis of the expression of PEP‐dependent genes in WT and *osppr16* leaves at the (a) three‐leaf stage and (b) five‐leaf stage. *OsTPI* was used as reference gene. Error bars indicate SD (*n* = 3); *, *P* < 0.05; **, *P* < 0.01 (Student’s *t*‐test) in (b); all *P* < 0.01 in (a). (c) Immunoblot analysis of selected proteins encoded by PEP‐dependent genes in WT and *osppr16* leaves at the three‐leaf stage. Samples of 25 μg total cellular protein extracted from *osppr16d* leaves were loaded and compared with a dilution series of WT protein extracts. The heat shock protein HSP82 was used as an internal control. (d) Immunoblot analysis of the same proteins at the five‐leaf stage. Samples of 25 μg total protein from WT and *osppr16d* leaves were analyzed by Western blot analysis. HSP82 was used as an internal control.

The accumulation of RpoB, the β‐subunit of PEP, was reduced in the absence of editing at *rpoB*‐545 in *osppr16* mutants. To determine the consequences on PEP activity and transcription of the chloroplast genome, the expression of PEP‐dependent genes was analyzed by qRT‐PCR. In *osppr16* mutant plants, the expression of PEP‐dependent genes such as *atpB*, *psaA*, *psbB* and *rbcL* was strongly reduced (to < 20% of the expression in the WT) at the three‐leaf stage (Fig. 7a). Interestingly, at the five‐leaf stage, the expression of most PEP‐dependent genes in *osppr16* mutant plants had nearly fully recovered and was similar to that in the WT, with the exception that the expression of *petN* was slightly lower than in the WT (Fig. 7b). Immunoblot analysis of the accumulation of proteins encoded by PEP‐dependent genes confirmed the significance of the reduced mRNA levels at the level of the protein products (Fig. 7c,d). Together, these results indicate that PEP activity in *osppr16* mutants is decreased during early leaf development but recovers at later developmental stages.

### Low levels of tRNA^Glu^ synthesis in *osppr16* during early leaf development

**Fig. 8:**
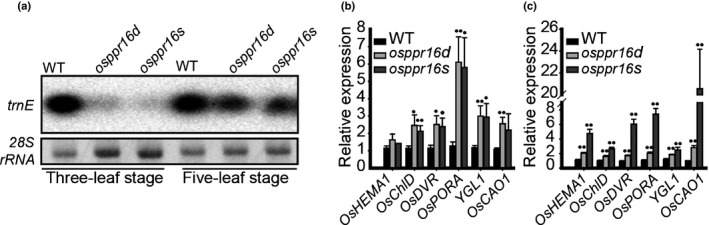
Expression of Chl biosynthesis‐related genes in rice (*Oryza sativa*) wild‐type (WT) and *osppr16* mutant plants. (a) Northern blot analysis of accumulation of tRNA^Glu^ in *osppr16* mutants and WT plants at the three‐leaf stage and five‐leaf stage. *28S rRNA* accumulation was used as internal loading control. (b, c) Quantitative real‐time PCR analysis of the expression of nuclear genes related to tetrapyrrole synthesis at the (b) three‐leaf stage and (c) five‐leaf stage. *OsTPI* was used as reference gene. Error bars indicate SD (*n* = 3); *, *P* < 0.05; **, *P* < 0.01 (Student’s *t*‐test).

tRNA^Glu^ encoded by the chloroplast *trnE* gene is unique, in that it is not only required for chloroplast translation but also serves as substrate for Chl synthesis. In the committed step of the pathway, the enzyme glutamyl‐tRNA reductase (GluTR) utilizes charged tRNA^Glu^ to synthesize the universal precursor for the biosynthesis of all tetrapyrroles: 5‐aminolevulinic acid (Beale, 1999). To explore the possibility that tRNA^Glu^ provision limits Chl biosynthesis in *osppr16* mutant plants, and thus is causally responsible for the pigment‐deficient phenotype, *trnE* gene expression was investigated by Northern blot analysis. Interestingly, at the three‐leaf stage, accumulation of tRNA^Glu^ in *osppr16* mutant plants was very low (Fig. 8a). By contrast, at the five‐leaf stage, tRNA^Glu^ accumulation in the mutants had recovered and nearly reached WT levels (Fig. 8a). As controls, the accumulations of *trnN* and *rpl23* were analyzed. *trnN* is a PEP and NEP‐dependent gene, according to the analysis of its promoter (Fig. S6a). The accumulation of *trnN* in *osppr16* mutant plants was similar to that in the WT at the three‐leaf stage and even higher than in the WT at the five‐leaf stage (Fig. S6b). *rpl23* is a NEP‐dependent gene (Zhelyazkova *et al*., 2012), and the accumulation of *rpl23* transcripts in *osppr16* mutant plants was higher than in the WT at the three‐leaf and five‐leaf stages (Fig. S6c). Together, these results suggest that tRNA^Glu^ synthesis in *osppr16* mutants is very strongly affected by the PEP deficiency in early leaf development.

To further explore the Chl deficiency in the mutants, the expression of key Chl synthesis genes (*OsHEMA1*, *OsChlD*, *OsDVR*, *OsPORA*, *YGL1* and *OsCAO1*) was analyzed by qRT‐PCR. In the *osppr16* mutants, all genes were found to be significantly upregulated at both the three‐leaf stage and the five‐leaf stage compared with WT plants (with the exception of *OsHEMA1*, which showed a significant increase only at the five‐leaf stage; Fig. 8b,c). These results suggest that, in *osppr16* mutants, the expression of nuclear genes for components of the tetrapyrrole biosynthetic pathway responds to the Chl deficiency in the chloroplast.

### *osppr16* mutants display a *gun* phenotype

Expression of a large set of nuclear genes encoding plastid‐related functions is regulated by retrograde signals. Some retrograde signals emanate from developing chloroplasts (Singh *et al*., 2015) and control the expression of nuclear genes at the transcriptional and post‐transcriptional levels (Wu *et al*., 2019b, 2019). The genetic basis of retrograde signaling was revealed by the isolation of *Arabidopsis* mutants with disrupted retrograde signaling. These mutants were dubbed *genomes uncoupled* (*gun*) mutants, and most of them are related to genes involved in tetrapyrrole biosynthesis (Susek *et al*., 1993; Mochizuki *et al*., 2001; Larkin *et al*., 2003; Wu *et al*., 2018, 2019a). To explore whether retrograde signaling is affected in *osppr16* mutants and determine whether the mutants display a *gun* phenotype at the molecular level, we analyzed the expression of *OsLHCB2* and *OsRBCS2*, two classical target genes of GUN‐dependent retrograde signals. As a negative control, the expression of *OsPPI2*, a gene not regulated by plastid retrograde signals, was also analyzed. To visualize retrograde regulation, *osppr16* and WT seedlings were grown in the presence or absence of NF at the three‐leaf stage. In the absence of NF, the expression levels of *OsLHCB2* and *OsRBCS2* were very similar in *osppr16* mutants and the WT (Fig. S7a,b). By contrast, the expression of *OsLHCB2* and *OsRBCS2* in *osppr16* mutants was higher (i.e. less repressed) than in the WT in the presence of NF (Fig. S7a,b). Expression of the control gene *OsPPI2* showed no significant difference between *osppr16* and the WT, neither in the absence nor in the presence of NF (Fig. S7c). Thus, *osppr16* mutants appear to display a molecular *gun* phenotype that is suggestive of disturbed plastid retrograde signaling. Although the precise role of *OsPPR16* in retrograde communication needs to be investigated further, it seems conceivable that OsPPR16 acts through its effects on the tetrapyrrole biosynthesis pathway and/or the plastid gene expression pathway of plastid retrograde signaling.

## Discussion

In the course of this work, we have characterized a rice chloroplast RNA editing factor that acts in a striking developmental stage‐specific fashion. OsPPR16 is a chloroplast‐localized PLS‐DYW subfamily PPR protein (Fig. 3). We have shown that *rpoB*‐545 editing is undetectable in both pale and green leaves of *osppr16* knockout mutants (Fig. 4a), whereas all other 23 chloroplast editing sites, including *rpoB*‐467 and *rpoB*‐560, are edited normally (Fig. S4). Complementation studies proved that *rpoB*‐545 editing in *osppr16* can be restored by transgenic expression of *OsPPR16* (Fig. 4e). Furthermore, knockdown of *OsPPR16* leads to reduced editing efficiency at *rpoB*‐545 (Fig. 4b,c).

Previous studies have reported 21 PPR and PPR‐related proteins that are required in plastid RNA editing in *Arabidopsis* (Lu, 2018). Two PPR proteins (OsPPR4 and OsPPR6) have been shown to participate in editing of a single plastid RNA editing site in rice (Asano *et al*., 2013; Tang *et al*., 2017). In *Arabidopsis*, the expression of CRR22, which shares 57.7% amino acid identity with OsPPR16, is required for RNA editing of *rpoB*‐551, as well as of *ndhB*‐746 and *ndhD*‐887 (Okuda *et al*., 2009). In rice, the editing sites *ndhB*‐746 and *ndhD*‐887 are lost due to cytosine‐to‐thymine mutations at the DNA level. Thus, *rpoB*‐545 (corresponding to *rpoB*‐551 in *Arabidopsis*) remains as the only RNA editing target (Fig. S3). Phylogenetic analysis of OsPPR16 indicates that OsPPR16 homologues are conserved in many vascular plants but are absent from the moss *Physcomitrella patens*, in which none of the three editing sites (*rpoB*‐545, *ndhB*‐746 and *ndhD*‐887) is present (Fig. S3).

Although in *Arabidopsis* the editing of *rpoB*‐545, *ndhB*‐746 and *ndhD*‐887 is impaired in *crr22* loss‐of‐function mutants, the mutant plants do not show an obvious phenotype under standard growth conditions (Okuda *et al*., 2009). In rice *osppr16* mutants, only the editing at *rpoB*‐545 is defective, which, however, severely affects chloroplast development during early leaf development. The reason for this striking phenotypic difference between the *Arabidopsis* and rice mutants is currently unclear. A possible explanation could be that PEP activity plays a more important role in early chloroplast development in rice than in *Arabidopsis*. Alternatively, the edited event could be more important for the function of RpoB in rice than in *Arabidopsis*. Our findings reported here indicate that studies in a single species do not necessarily permit general conclusions about the functional relevance of RNA editing events in plant organelles.

In the *Arabidopsis ys1* mutant, lack of editing at another site in the *rpoB* mRNA, *rpoB*‐338 (corresponding to position *rpoB*‐332 in rice, which does not undergo RNA editing, because a T is encoded at the DNA level) leads to a virescent phenotype at early leaf development that disappears 3 wk after germination (Zhou *et al*., 2009). This phenotype is similar to that of our *osppr16* mutants and may suggest that high PEP activity is also crucial to early leaf development in *Arabidopsis*.

Although the *rpoB* mRNA level was increased in *osppr16* mutants, the accumulation of the encoded β‐subunit of PEP was severely decreased at the three‐leaf stage (Fig. 5). Consequently, the expression of PEP‐dependent genes in *osppr16* mutant plants was strongly affected at the three‐leaf stage (Fig. 7a), but virtually restored in the green leaves at the five‐leaf stage (Fig. 7b). Conversely, the expression of NEP‐dependent genes was upregulated (Fig. 5b,c) in the mutants, which is a known consequence of PEP deficiency (Allison *et al*., 1996; Hajdukiewicz *et al*., 1997). Taken together, our results indicate that the lack of *rpoB* editing in *osppr16* mutants leads to low PEP activity, which limits chloroplast biogenesis in early leaf development, but is sufficient to sustain nearly normal levels of chloroplast gene expression at later stages of leaf development, when the biogenesis of the photosynthetic apparatus is completed and the demand for chloroplast RNA synthesis decreases. Our results do not entirely exclude the possibility that, through its RNA‐binding activity, OsPPR16 also directly regulates some other plastid genes, in addition to its function in RNA editing of *rpoB‐545*. Complementation of *osppr16* with an ‘edited’ *rpoB* allele (encoding a leucine residue at the DNA level) would be a suitable experiment to ultimately confirm that the editing function of OsPPR16 is solely responsible for the greening defect observed in the *osppr16* mutant. However, in the absence of a chloroplast transformation technology for rice, this experiment is currently not technically feasible.

**Fig. 9:**
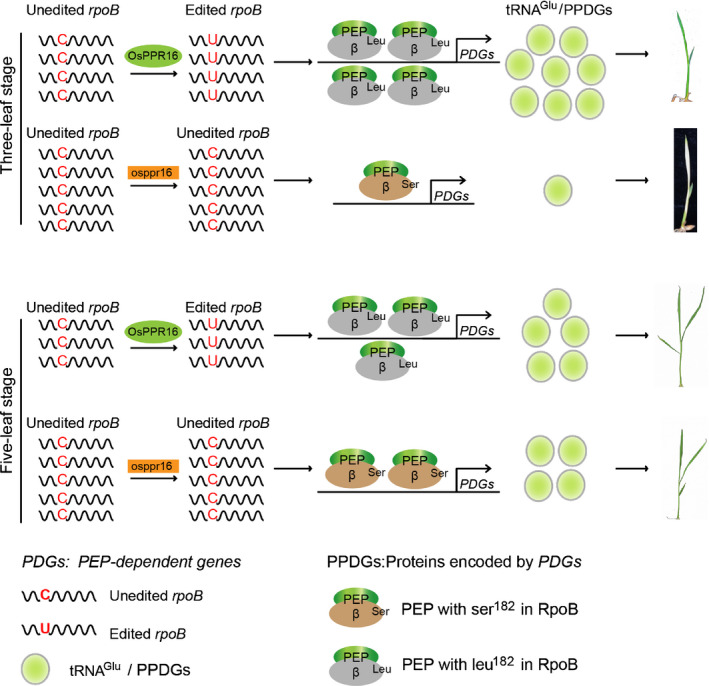
A working model for the role of OsPPR16 during chloroplast development in rice. During early chloroplast development, OsPPR16 is particularly important to edit *rpoB* transcripts to ensure maximum RpoB accumulation, thereby producing an active plastid‐encoded RNA polymerase (PEP) pool that is capable of efficiently transcribing PEP‐dependent genes (*PDG*s). The accumulation of tRNA^Glu^ and proteins encoded by other PEP‐dependent genes is required to meet the demand for Chl and protein synthesis during the rapid development of proplastids into chloroplasts. Loss of OsPPR16 leads to an extremely low level of tRNA^Glu^ (and proteins encoded by other PEP‐dependent genes, PPDGs), thereby limiting Chl biosynthesis and protein synthesis (and, potentially, also protein stability in the absence of sufficient pigments for incorporation into Chl‐binding proteins) in the developing chloroplasts, thus causing the pale phenotype during early leaf development. Lower demands for PEP activity at later developmental stages and gradually increasing PEP accumulation in the mutants alleviate the phenotype and facilitate leaf greening. See the Discussion section for more details.

In addition to participating in plastid protein synthesis, tRNA^Glu^ is also the precursor for Chl synthesis (Beale, 1999). Thus, decreased accumulation of tRNA^Glu^ can affect Chl synthesis and chloroplast biogenesis (Zhou *et al*., 2009; Börner *et al*., 2015). Consistent with such a limiting role, tRNA^Glu^ accumulation in *osppr16* mutants was found to be extremely low at the three‐leaf stage (Fig. 8a). Nearly full recovery at the five‐leaf stage corresponded well with the increase in Chl content and may suggest that the pattern of tRNA^Glu^ accumulation in leaf development explains the developmental phenotype of the *osppr16* mutant plants. We therefore propose a model (Fig. 9) in which active OsPPR16 expression in young leaves (Fig. S8) is required to edit *rpoB* transcripts, thus ensuring efficient RpoB protein accumulation. This leads to production of a sufficiently large and active pool of PEP that is capable of transcribing PEP‐dependent genes to levels that are sufficient to satisfy the high demands of the chloroplast biogenesis program. The accumulation of tRNA^Glu^ is required to meet the large demand for Chl synthesis during the rapid development of proplastids and etioplasts to chloroplasts. At the same time, high PEP activity is also needed to synthesize Chl‐binding proteins (photosystem subunits), many of which are also encoded by PEP‐dependent genes. Loss of OsPPR16 leads to an extremely low level of tRNA^Glu^ (and proteins encoded by other PEP‐dependent genes), thereby limiting Chl synthesis in the developing chloroplasts and leading to the pigment‐deficient phenotype during early leaf development. In the WT, the level of RpoB decreases at the five‐leaf stage, in response to the decreasing demands of plastid gene expression, now that chloroplast development is largely completed. By contrast, the accumulation of RpoB in *osppr16* mutants increases over time to gradually overcome the PEP deficiency. Consequently, the accumulation of tRNA^Glu^ and proteins encoded by other PEP‐dependent genes increases, ultimately reaching levels that are similar to those in the WT and sufficient to facilitate leaf greening.

In conclusion, our study suggests that *OsPPR16* is needed for the efficient accumulation of RpoB by specifically editing site *rpoB*‐545. In particular, the high demand for Chl synthesis during early leaf development requires high PEP activity (for *trnE* gene expression) and provides a likely molecular explanation of the striking developmental phenotype of the rice *osppr16* mutants. Our work highlights interesting differences in the function of editing PPRs in monocots vs dicots and links RNAP deficiency to a specific molecular process (tetrapyrrole biosynthesis) that becomes limiting in the chloroplast developmental program.

## Author contributions

FZ, YL and WH conceived the project and designed the experiments. WH performed most of the experiments, assisted by Yongli Zhu, CZ and HC. Yang Zhang performed the qRT‐PCR analysis. LS and Yu Zhang performed the protein structural analysis. QF and CM performed the subcellular localization analyses. QL performed the splicing analyses. CG and Yong Zhou performed the bioinformatics analyses. JZ helped with the Northern blot. WH prepared all the figures and wrote the manuscript with contributions from FZ and RB. All the authors read and approved the final manuscript.

## Supporting information

Please note: Wiley Blackwell are not responsible for the content or functionality of any Supporting Information supplied by the authors. Any queries (other than missing material) should be directed to the *New Phytologist* Central Office.

**Fig. S1** Phenotype of rice (*Oryza sativa*) *osppr16* mutant and WT plants at the tillering stage.
**Fig. S2** Alignment of the amino acid sequences of OsPPR16 and its closest homologues from *Arabidopsis thaliana* (NP_172596.1, CRR22), *Nicotiana tabacum* (XP_016505961.1) and *Zea mays* (NP_001356569.1).
**Fig. S3** Phylogenetic analysis of OsPPR16 and the nucleotide positions corresponding to the editing sites *rpoB*‐545, *ndhB*‐746 and *ndhD*‐887 in 79 land plants.
**Fig. S4** Editing efficiency of 24 plastid RNA editing sites in rice (*Oryza sativa*) WT and *osppr16* mutant plants.
**Fig. S5** Splicing analyses of rice chloroplast transcripts in rice (*Oryza sativa*) WT and *osppr16d* mutant plants at the three‐leaf stage.
**Fig. S6** Expression of *trnN* and *rpl23* in rice (*Oryza sativa*) WT and *osppr16* mutant plants.
**Fig. S7** *osppr16* mutants show a *gun* phenotype on norflurazon.
**Fig. S8** Tissue‐specific expression profiles of *OsPPR16* in rice (*Oryza sativa*) plants.
**Methods S1** Complementation of the *osppr16* mutant.
**Table S1** List of oligonucleotides used in this study.
**Table S2** Accession numbers of RpoB, NdhB, NdhD and homologous of OsPPR16 in 79 land plants.
**Table S3** Genotype and phenotype of T_0_ transgenic plants.
