# Supplementary material for: Accumulation of the RNA polymerase subunit RpoB depends on RNA editing by OsPPR16 and affects chloroplast development during early leaf development in rice
Source: New Phytol. 2020 Jul 22;228(4):1401–16. doi: 10.1111/nph.16769 (PMC7689822; doi:10.1111/nph.16769)
Supplement: Supplementary file 1 — Fig. S1 Phenotype of rice (Oryza sativa) osppr16 mutant and WT plants at the tillering stage. Fig. S2 Alignment of the amino acid sequences of OsPPR16 and its closest homologues from Arabidopsis thaliana (NP_172596.1, CRR22), Nicotiana tabacum (XP_016505961.1) and Zea mays (NP_001356569.1). Fig. S3 Phylogenetic analysis of OsPPR16 and the nucleotide positions corresponding to the editing sites rpoB‐545, ndhB‐746 and ndhD‐887 in 79 land plants. Fig. S4 Editing efficiency of 24 plastid RNA editing sites in rice (Oryza sativa) WT and osppr16 mutant plants. Fig. S5 Splicing analyses of rice chloroplast transcripts in rice (Oryza sativa) WT and osppr16d mutant plants at the three‐leaf stage. Fig. S6 Expression of trnN and rpl23 in rice (Oryza sativa) WT and osppr16 mutant plants. Fig. S7 osppr16 mutants show a gun phenotype on norflurazon. Fig. S8 Tissue‐specific expression profiles of OsPPR16 in rice (Oryza sativa) plants. Methods S1 Complementation of the osppr16 mutant. Table S1 List of oligonucleotides used in this study. Table S2 Accession numbers of RpoB, NdhB, NdhD and homologous of OsPPR16 in 79 land plants. Table S3 Genotype and phenotype of T0 transgenic plants. [file NPH-228-1401-s001.pdf]

New Phytologist Supporting Information Figs S1–S8, Tables S1-S3, and Methods S1

Article title: Accumulation of the RNA polymerase subunit RpoB depends on RNA editing by OsPPR16 and affects chloroplast development during early leaf development in rice
Authors: Weifeng Huang, Yang Zhang, Liqiang Shen, Qian Fang, Qun Liu, Chenbo Gong, Chen Zhang, Yong Zhou, Cui Mao, Yongli Zhu, Jinghong Zhang, Hongping Chen, Yu Zhang, Yongjun Lin, Ralph Bock, Fei Zhou
Article acceptance date: 11 June 2020

The following Supporting Information is available for this article:

Fig. S1 Phenotype of rice (*Oryza sativa*) *osppr16* mutant and WT plants at the tillering stage.

Fig. S2 Alignment of the amino acid sequences of OsPPR16 and its closest homologues from *Arabidopsis thaliana* (NP_172596.1, CRR22), *Nicotiana tabacum* (XP_016505961.1), and *Zea mays* (NP_001356569.1).

Fig. S3 Phylogenetic analysis of OsPPR16 and the nucleotide positions corresponding to the editing sites *rpoB*-545, *ndhB*-746, and *ndhD*-887 in 79 land plants.

Fig. S4 Editing efficiency of 24 plastid RNA editing sites in rice (*Oryza sativa*) WT and *osppr16* mutant plants.

Fig. S5 Splicing analyses of rice chloroplast transcripts in rice (*Oryza sativa*) WT and *osppr16d* mutant plants at the three-leaf stage.

Fig. S6 Expression of *trnN* and *rpl23* in rice (*Oryza sativa*) WT and *osppr16* mutant plants.

Fig. S7 *osppr16* mutants show a gun phenotype on Norflurazon.

Fig. S8 Tissue-specific expression profiles of *OsPPR16* in rice (*Oryza sativa*) plants.

Table S1 List of oligonucleotides used in this study.

Table S2 Accession numbers of RpoB, NdhB, NdhD, and homologous of OsPPR16 in 79 land plants.

Table S3 Genotype and phenotype of T_0 transgenic plants.

Methods S1 Complementation of the *osppr16* mutant.

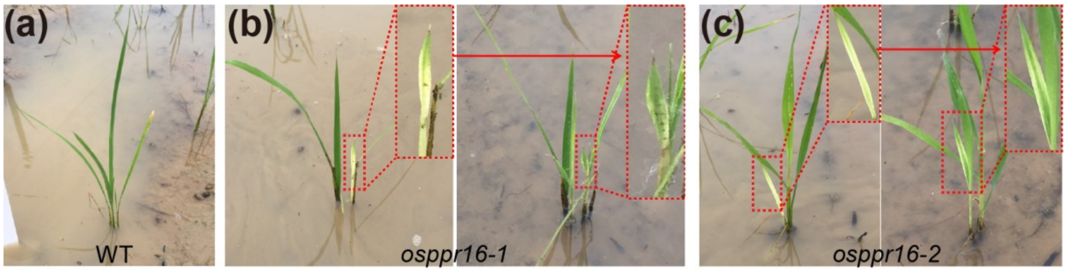

Fig. S1 Phenotype of rice (*Oryza sativa*) *osppr16* mutant and WT plants at the tillering stage. (a) WT plant. (b) *osppr16* mutant plant obtained with Strategy I. (c) *osppr16* mutant generated by Strategy II (cf. Fig. 1).

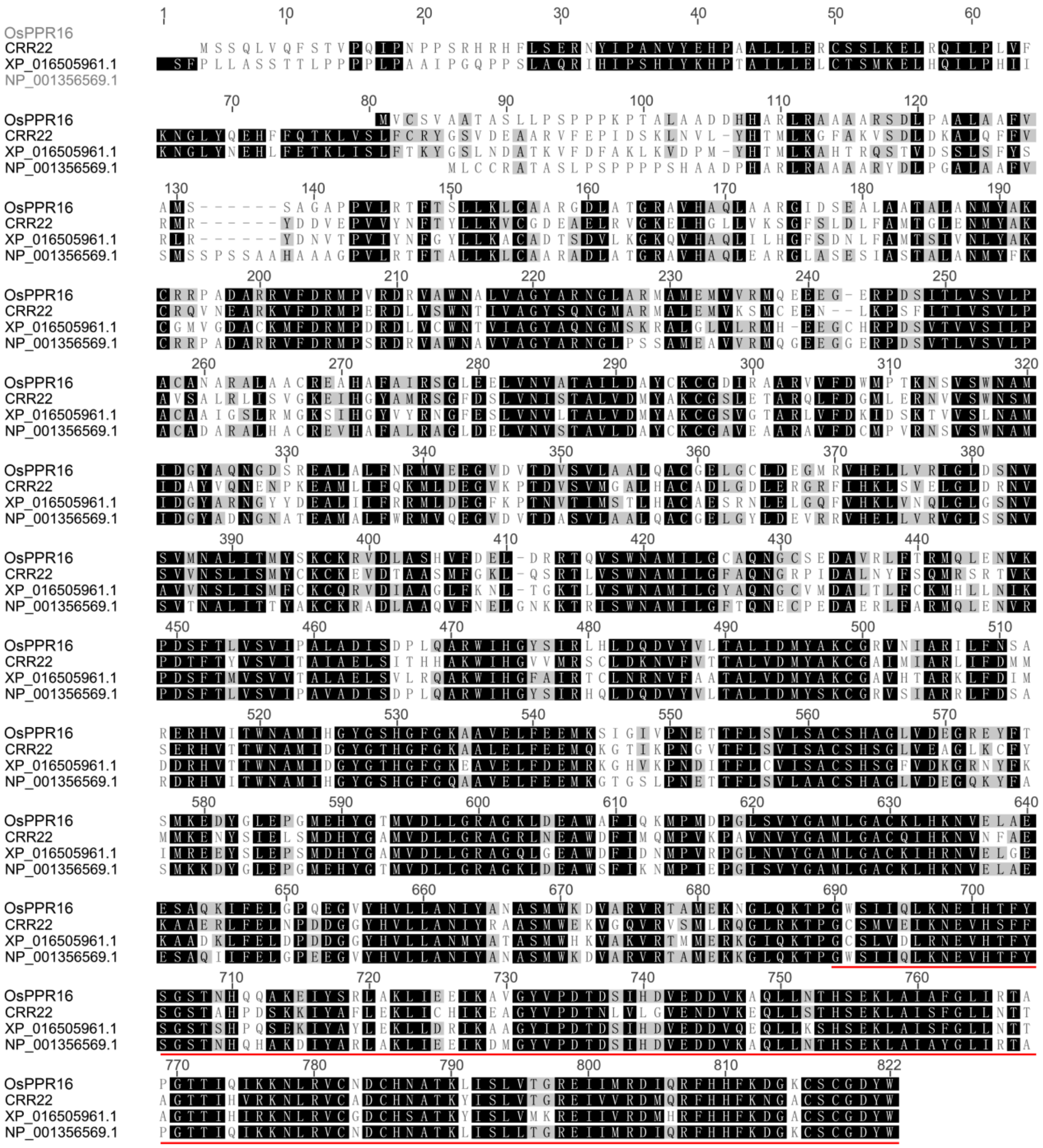

Fig. S2 Alignment of the amino acid sequences of OsPPR16 and its closest homologues from *Arabidopsis thaliana* (NP_172596.1, CRR22), *Nicotiana tabacum* (XP_016505961.1), and *Zea mays* (NP_001356569.1). The DYW domain is underlined in red.

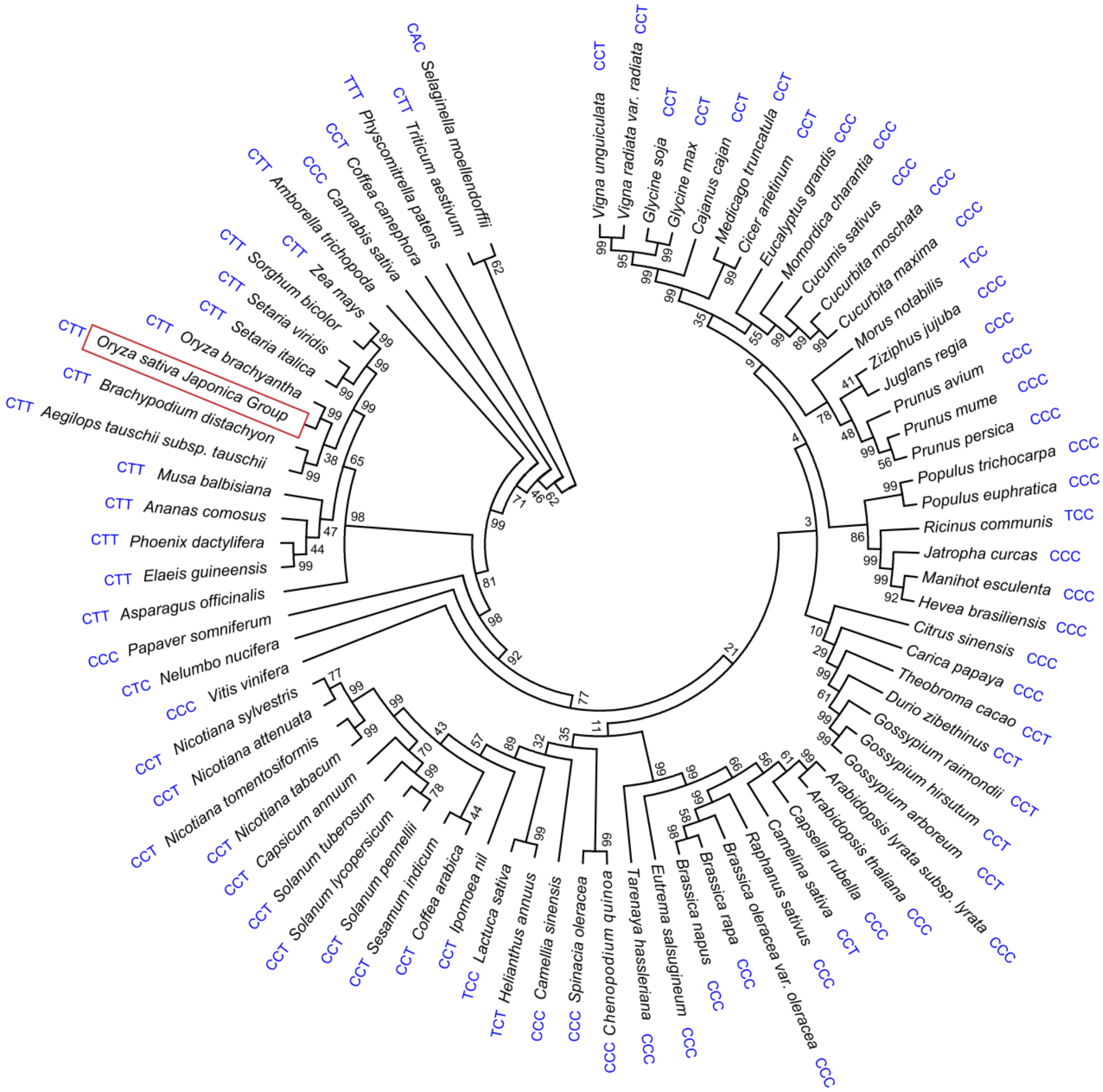

Fig. S3 Phylogenetic analysis of OsPPR16 and the nucleotide positions corresponding to the editing sites *rpoB*-545, *ndhB*-746 and *ndhD*-887 in 79 land plants. All protein sequences were downloaded from NCBI, and the phylogenetic tree was constructed by the MEGA 5.0 software using the NJ method with 1000 bootstrap replicates. The nucleotides corresponding to the three editing sites *ropB*-545, *ndhB*-746, and *ndhD*-887 are given in blue. *Oryza sativa Japonica* group was highlighted in the red box.

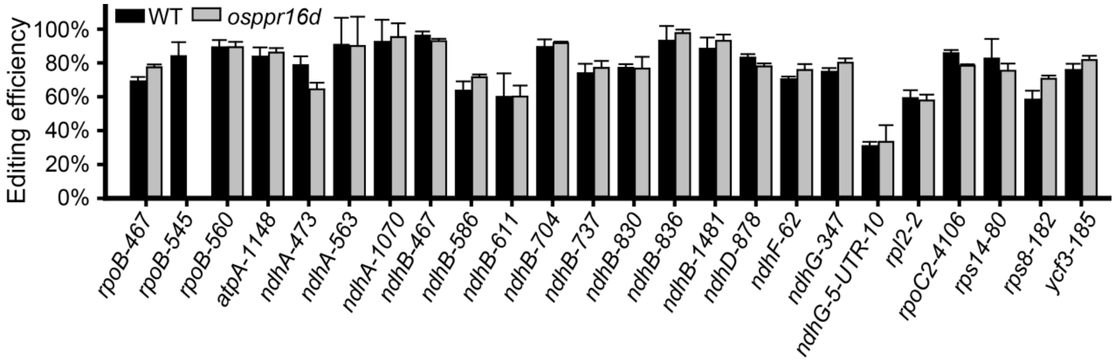

Fig. S4 Editing efficiency of 24 plastid RNA editing sites in rice (*Oryza sativa*) WT and *osppr16* mutant plants. Error bars indicate SD (n = 3).

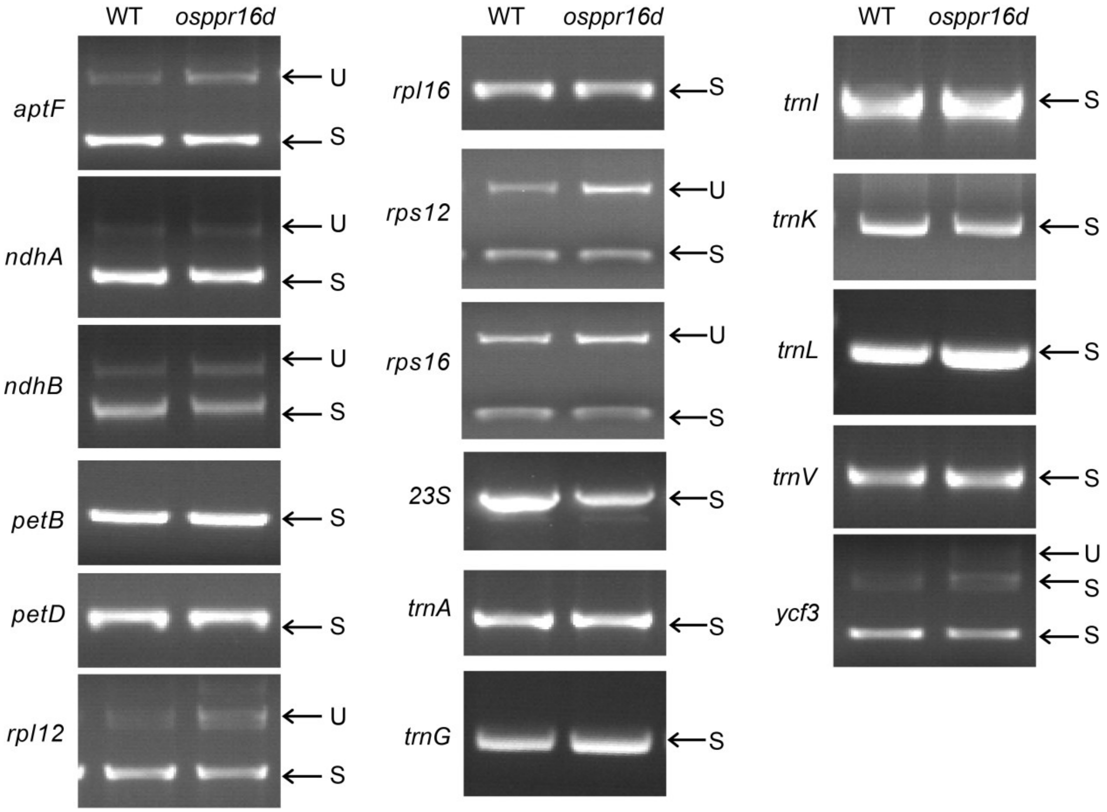

Fig. S5 Splicing analyses of rice chloroplast transcripts in rice (*Oryza sativa*) WT and *osppr16d* mutant plants at the three-leaf stage. Splicing efficiency was comparatively assessed by RT-PCR. Transcripts are indicated at the left. Spliced (S) and unspliced (U) transcripts are marked at the right.

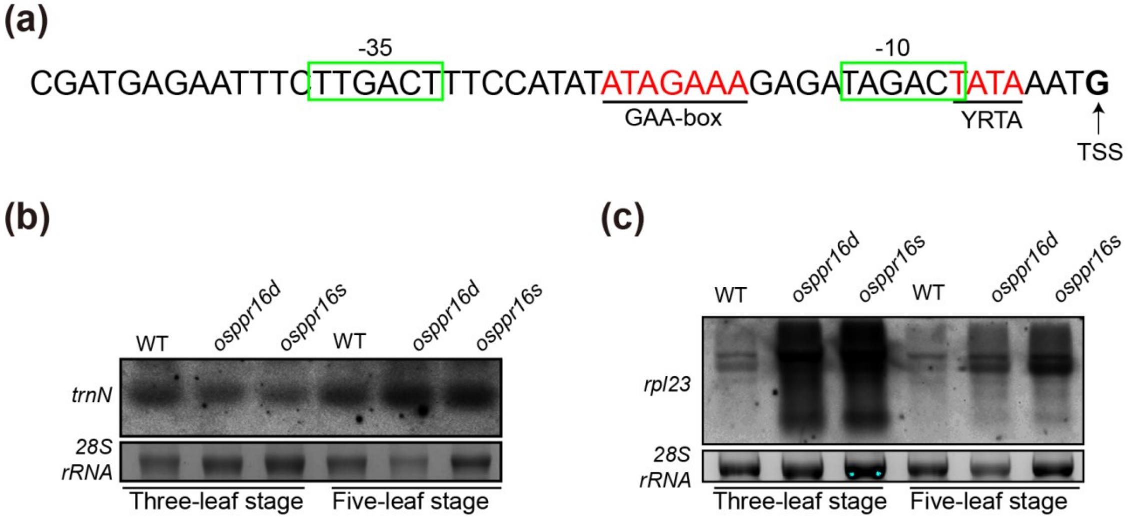

Fig. S6 Expression of *trnN* and *rpl23* in rice (*Oryza sativa*) WT and *osppr16* mutant plants. (a) Motif analysis of the *trnN* promoter. The -35 and -10 boxes (in green box) are typical motifs of PEP promoters, the GAA and YRTA motifs (red text) are typical motifs of NEP promoters (Liere and Börner, 2007). TTS: transcription start site. (b, c) Northern blot analysis to determine accumulation of *trnN* (b) and *rpl23* (c) transcripts in *osppr16* mutants and WT plants at the three-leaf stage and the five-leaf stage. *28S rRNA* accumulation is shown as internal loading control.

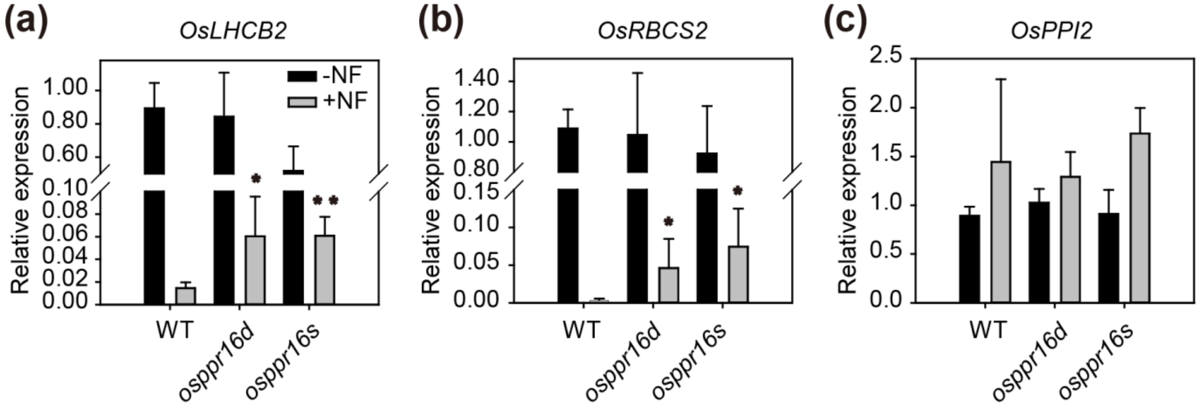

Fig. S7 *osppr16* mutants show a *gun* phenotype in the presence of norflurazon (NF). (a-c) qRT-PCR analysis of the expression of *OsLHCB2* (a), *OsRBCS2* (b), and *OsPPI2* (c) in rice seedlings grown on half-strength MS medium under white light with or without 5 µM NF for 7 d. *OsPPI2* was used as reference gene that is not controlled by retrograde signals. Error bars indicate the SD

(n = 2-4), * indicates P < 0.05, ** indicates P < 0.01. The asterisks denote a significant difference to the WT in the same treatment (-NF or +NF; Student's *t*-test).

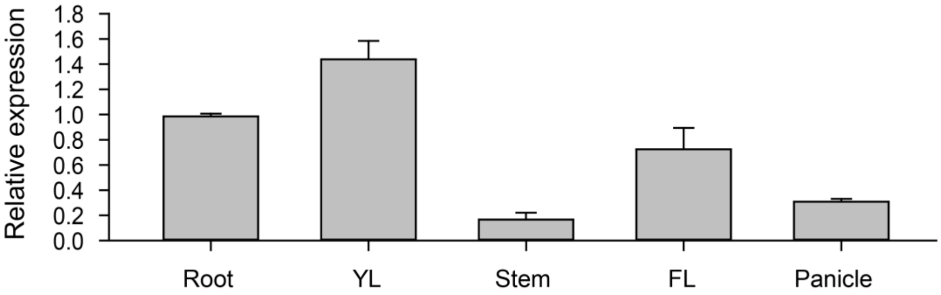

Fig. S8 Tissue-specific expression profiles of *OsPPR16* in rice (*Oryza sativa*) plants. qRT-PCR was used to determine the expression levels of *OsPPR16* in different tissues. *OsTPI* was used as reference gene. The expression level of *OsPPR16* in roots was set as 1.0. YL: young leaves; FL: flag leaves. Error bars indicate SD (n = 3).

Table S1 List of oligonucleotides used in this study.

**Primer**	**Sequence (5'-3')**	**Application**
Target1-F	GGCACGCGCGGAACGGCCTCGCC	Plasmid construction
Target1-R	AAACGGCGAGGCCGTTCCGCGCG	Plasmid construction
Target2-F	GCCGGCCGCGCGCTCCGACCTGC	Plasmid construction
Target2-R	AAACGCAGGTCGGAGCGCGCGGC	Plasmid construction
Target3-F	GCCGGATATTTCAGATCCTCTGC	Plasmid construction
Target3-R	AAACGCAGAGGATCTGAAATATC	Plasmid construction
P1	AGTGGCGGTTGCATGTAGAG	Genotype analysis
P2	ATCAAGACAGCCAAGCTCCC	Genotype analysis
P3	GGGAGCTTGGCTGTCTTGAT	Genotype analysis
P4	CGGCCAAGAAGATCCACCAT	Genotype analysis
RNAi16-F	ACTAGTGGTACCTTGCAAGGGTGAG AACTGCT	Plasmid construction
RNAi16-R	GAGCTCGGATCCGAGCCCAAAAGCA ATGGCAA	Plasmid construction
CO16A-F	GCATTTCTTCCTAGCTCAACCA	Plasmid construction
CO16A-R	GTCGACTCAGCATGGTTCAAAATCG CA	Plasmid construction
CO16B-F	CATGATTACGAATTCGAGCTCAAAG TAAAGTGCGGGGATCG	Plasmid construction

CO16B-R	CTATGTAAATTATATTAGCTGAGCT CTAGATTTAGAAGAAT	Plasmid construction
Cas9-F	GCATGAAGAGGATCGAGGAG	Genotype analysis
Cas9-R	GATCTCTTGCTCGGACTTGG	Genotype analysis
T-DNA16-F	CTAGCCAATACGCAAACCGC	Genotype analysis
T-DNA16-R	AAGGCCCTATTTGTCCCACG	Genotype analysis
Sbu16YPF-F	GGGGACAAGTTTGTACAAAAAAGC AGGCTTCACCATGGTGTGCTCCGTG GCTGC	Plasmid construction
Sbu16YPF-R	GGGGACCACTTTGTACAAGAAAGCT GGGTGCCAGTAGTCTCCACATGAGC	Plasmid construction
trnE-F	CCCCTATCGTCTAGTGGTTCAG	Northern blot
trnE-R	TACCCCCAGGGGAAGTCG	Northern blot
trnN-F	TCCTCAGTAGCTCAGTGGTAGA	Northern blot
trnN-R	GGATTCGAACCTACGACCAGT	Northern blot
rpl23-F	TGGATGGAATCAAATACGCAGT	Northern blot
rpl23-R	GGGCCCATTCTTCTACCCTT	Northern blot
atpF-F	TTTTAGCTCACTGGCCATCC	RNA splicing
atpF-R	TTCATCGCCCTTTGTTTTTC	RNA splicing
ndhA-F	ATGATAATAGACAGGGTACAGG	RNA splicing
ndhA-R	TTATAGTGAAACAAGTTGGGAAG	RNA splicing
ndhB-F	ATGATCTGGCATGTACAGAATG	RNA splicing
ndhB-R	CTAAAAGAGGGTATCCTGAGCA	RNA splicing
petB-F	TTCTCATATACGGTTCTCGG	RNA splicing
petB-R	TAAAGGGCCCGAAATACCTT	RNA splicing
petD-F	ATGGGAGTAACAAAGAAACC	RNA splicing
petD-R	TGTTGCTCCAATACCTAACC	RNA splicing
rpl2-F	ACGGCGAAACATTTATACAA	RNA splicing
rpl2-R	TTACTTACGGCGACGAAGAATA	RNA splicing
rpl16-F	ATGCTTAGTCCCAAAAGAAC	RNA splicing
rpl16-R	AACCGAAGAAATTGACTTCG	RNA splicing
rps12-F	ACTATCAACCCCAAAAAACC	RNA splicing
rps12-R	TTTGGCTTTTTGACCCCAT	RNA splicing
rps16-F	AAAACGATGTGGTAGAAAGC	RNA splicing
rps16-R	AGAATTCCGCCTTCCTTAAA	RNA splicing
trnA-F	GGGGATATAGCTCAGTTGGT	RNA splicing
trnA-R	TGGAGATAAGCGGACTCGAA	RNA splicing
trnG-F	TCGTTAGCTTGGAAGGCTAG	RNA splicing
trnG-R	GCGGGTATAGTTTAGTGGTA	RNA splicing

trnI-F	TGGGCCATCCTGGACTTGA	RNA splicing
trnI-R	AGCTCAGTGGTAGAGCGCG	RNA splicing
trnK-F	GGTTGCCCGGGACTCGAA	RNA splicing
trnK-R	GGGTTGCTAACTCAATGGTAGAG	RNA splicing
trnL-F	GGATATGGCGAAATCGGTA	RNA splicing
trnL-R	TGGGGATAGAGGGACTTGA	RNA splicing
trnV-F	TAGGGCTATACGGATTCGAA	RNA splicing
trnV-R	AGGGCTATAGCTCAGTTCGG	RNA splicing
ycf3-F	ATGCCTAGATCCCGTATAAATG	RNA splicing
ycf3-R	TTATTCAAATTCAAAGCGCTTC	RNA splicing
23S-F	TTCAAAAGAGGAAAGGCTTG	RNA splicing
23S-R	AGAGAGCACTCATCTTGGGG	RNA splicing
atpA-F	GTAGCTGAAATGGCGGATTC	RNA editing
atpA-R	ATCTGCTCTTCCACTGGAAG	RNA editing
atpF-F	CGTCCTATCTATAAGAGGAGAGC	RNA editing
atpF-R	CATTCGATACTCATCCGCTTC	RNA editing
ndhA-F	TGTTTGTTGACCCATGAACCC	RNA editing
ndhA-R	TTTATGCAAGAGTGGAAGCCC	RNA editing
ndhB-1F	GGAAATAGCATGGAATAAGGTTTA	RNA editing
ndhB-1R	TTCCTGGGGAGTTATACATTTGTG	RNA editing
ndhB-2F	ATTTACTCATGGGTGGGGCA	RNA editing
ndhB-a	TCCTTCGTAGACGTCAGGAG	RNA editing
ndhB-b	CCCACTCCAGTCGTTGCTTT	RNA editing
ndhB-2R	ATTACCCTAGCGCCCATTCC	RNA editing
ndhD-F	CCGCAGCATGGCTCTATCTT	RNA editing
ndhD-R	TCTATCACAAGCCGTTCCCG	RNA editing
ndhF-F	GAATTAGTTTGGTGAATCGG	RNA editing
ndhF-R	GGAACTAGTAACCAATCCCAAC	RNA editing
ndhG-F	CGAATACCTACTGCTTATTGC	RNA editing
ndhG-R	TCGGTTGCTAAATGAATCCC	RNA editing
petB-F	TGACGCGATTCCGGTAATAG	RNA editing
petB-R	GAAATACCACAACCTACCCG	RNA editing
psbE-F	GTCAGTACGGGTTTAGCTTA	RNA editing
psbL-R	CCAGTATAGACTGGTACGAT	RNA editing
rpl2-F	GGTGTTAAGGTAGTAGCTGT	RNA editing
rpl2-R	CCTCTATGCCTTGCGGTAATG	RNA editing
rpl20-F	ACCCATGAACCCAGTTACCA	RNA editing
rpl20-R	AATGGGAAAAAGGATGGCTAA	RNA editing
rpoA-F	GAAAGAACAATGGGGAAGAAAA	RNA editing
rpoA-R	AGATGCTTCTCTAGAGTGTC	RNA editing
rpoB-F	GGATGCTGTGTATGAATCAC	RNA editing

rpoB-R	CTTGGCTTGTTGTATCCGTC	RNA editing
rpoC1-F	ACGAGTACCTAATCACGAGG	RNA editing
rpoC1-R	TCCACAAGCACAAATTCTGC	RNA editing
rpoC2-F	GGCACTTTAGAGGAAGACTC	RNA editing
rpoC2-R	TACGACCTCGTAAGGCAGCT	RNA editing
rps2-F	CTCGGCAAAGCGTAAAGGTA	RNA editing
rps2-R	CTTCAAGATTGCCACATCTC	RNA editing
rps8-F	ACCTCTATAAGAAACGCGGACA	RNA editing
rps8-R	AGACTTCTCCCCCAATTCTGTT	RNA editing
rps14-F	CCATCTAGGATTAGAACCGT	RNA editing
rps14-R	ATCTTGTTGCACCCGGTAAC	RNA editing
ycf3-F	GGCTGAGCAGAAATACCTCT	RNA editing
ycf3-R	GTAATGACAGATCACGGCCA	RNA editing
Real16-F	TGTCAAAGCACAGCTGCTCA	qRT-PCR
Real16-R	TGGATTGTTGTGCCAGGGG	qRT-PCR
atpA-F	TCGCGGAGTTACAAGCCTTT	qRT-PCR
atpA-R	ATTCTCGTAATCGTCGGCCC	qRT-PCR
atpB-F	GGGGGAGCTGGAGTAGGTAA	qRT-PCR
atpB-R	CCCCTACTCCGCCAAATACG	qRT-PCR
atpE-F	ACTGACTCCTAAGCGAATT	qRT-PCR
atpE-R	GGCGTGGTTTGGTAATAC	qRT-PCR
atpH-F	CGTAGGTCTTGCTTCTAT	qRT-PCR
atpH-R	GTCTCGCAATACCTTCTA	qRT-PCR
atpI-F	ATTAGTCGTTGTTGTTCTTGT	qRT-PCR
atpI-R	GCTTGAATACCGCTTGTAA	qRT-PCR
petA-F	GCAGTGCTTCCCGATACTGT	qRT-PCR
petA-R	AGCACCCACATTCAACCCTC	qRT-PCR
petB-F	ACGTGGAAGTGCTAGTGTGG	qRT-PCR
petB-R	CGGCAGTAAGCAGAGGAAGT	qRT-PCR
petD-F	CCGACGGGCTTATTGACAGT	qRT-PCR
petD-R	GGTTGTAGCTACTGGGCGAC	qRT-PCR
petG-F	TTTCTATTTGGAATCGTCTTAGG	qRT-PCR
petG-R	ATCCCCACGTCTGTATTG	qRT-PCR
petL-F	CTAGTTATTTCGGTTTTCTATTGG	qRT-PCR
petL-R	GACGTATCTTGTTCAAGCC	qRT-PCR
petN-F	GGATATAGTAAGTCTCGCTTGG	qRT-PCR
petN-R	CTAGAGTCCACTCCTCCC	qRT-PCR
psaA-F	GCAGTCGGATGTTTGGGGTA	qRT-PCR
psaA-R	GAAATCTCGGAGCCACCCAT	qRT-PCR
psaB-F	ATGAACGGACACCTTTGGCT	qRT-PCR
psaB-R	CCCACCAATCTTGCTTGCAC	qRT-PCR

psaC-F	ACCGAAGATTGTGTGGGTTGT	qRT-PCR
psaC-R	AAGATAGAGCCATGCTGCGG	qRT-PCR
psaI-F	AACTTACCCTCTATTTTCGTGCC	qRT-PCR
psaI-R	TGCACATAAAGAAATAAAGAAGCC A	qRT-PCR
psaJ-F	CATATCTCTCTGTAGCACCCGT	qRT-PCR
psaJ-R	GGGAATGACAAAGCATCTGGG	qRT-PCR
psbA-F	TGTGGCCGCTCATGGTTATT	qRT-PCR
psbA-R	CCCTACTACAGGCCAAGCAG	qRT-PCR
psbB-F	GTTGAACAAGTAGGCGTAA	qRT-PCR
psbB-R	ACAGTAGCAGGATCAGAATA	qRT-PCR
psbC-F	GCTCCTTTAGGCTCTTTA	qRT-PCR
psbC-R	GTCGCTAACCAACTTCTA	qRT-PCR
psbD-F	GGCCCAATCGCTGTTTTTGT	qRT-PCR
psbD-R	CCAAAACTCGGCGCAAAGAA	qRT-PCR
psbE-F	CCCTATTCATTGCGGGTTGG	qRT-PCR
psbE-R	ACGGAATTCCTTGTCGGCTT	qRT-PCR
psbF-F	TTTTACAGTGCGATGGCTGG	qRT-PCR
psbF-R	CGTTGGATGAACTGCATTGCT	qRT-PCR
psbI-F	ACAGTAGTGATATTCTTTGTTTCCC	qRT-PCR
psbI-R	TCGTCACGCCCAGGATTA	qRT-PCR
psbJ-F	TGGCCGATACTACTGGAAGA	qRT-PCR
psbJ-R	CCCAATCCAGAATATGAACCGT	qRT-PCR
psbK-F	TTTCTTCGCCAAATTGCCCG	qRT-PCR
psbK-R	CAGCTTGCCAAACAAAGGCT	qRT-PCR
psbL-F	ATGACACAATCAAACCCGAATG	qRT-PCR
psbL-R	ACAAAAATGAGTAATAAACCCCAGT	qRT-PCR
psbM-F	ATATTCTCGCATTTATTGCTACTG	qRT-PCR
psbM-R	AATCATTTTGGCTGACTGTTTT	qRT-PCR
psbN-F	GGAAACAGCAACTTTAGTCG	qRT-PCR
psbN-R	CGTGTTCTTCGAATGGATCT	qRT-PCR
psbZ-F	GCTTCTCCTGATGGTTGGTCA	qRT-PCR
psbZ-R	AGCTACCAGAAAGACCAGTCC	qRT-PCR
rbcL-F	AGGGGAACGCGAAATGACTT	qRT-PCR
rbcL-R	AAAGATACCGCGAGCACGAT	qRT-PCR
rpoA-F	CCGCCTTCCGTAGAAATCGT	qRT-PCR
rpoA-R	CGCGATCTCTCTTGATCCGT	qRT-PCR
rpoB-F	GGCAGAAGAACTTGAGAA	qRT-PCR
rpoB-R	GGAAGAATAGGTGAGTGATT	qRT-PCR
rpoC1-F	CGTGCTACTTCTAATGTT	qRT-PCR
rpoC1-R	CCTGTATGCCTAATCTATG	qRT-PCR

rpoC2-F	GGCTGATGATGAGGTAAG	qRT-PCR
rpoC2-R	TGAAGAATAGAAGGATAGACAA	qRT-PCR
ycf4-F	ATTATATCTGTACTTCCATCTCA	qRT-PCR
ycf4-R	GTCATAACCACTGCCTAC	qRT-PCR
HEMA1-F	CGCTATTTCTGATGCTATGGGT	qRT-PCR
HEMA1-R	TCTTGGGTGATGATTGTTTGG	qRT-PCR
CHLD-F	GCTTGCAGAAAGCTACACAAGC	qRT-PCR
CHLD-R	AGGCCGTGAGCTAAAGGAGC	qRT-PCR
YGL1-F	ATGAACCTTACCGTCCTATTCCTT	qRT-PCR
YGL1-R	GGACCCACCAACAGCAAGAT	qRT-PCR
PORA-F	TGTACTGGAGCTGGAACAACAA	qRT-PCR
PORA-R	GAGCACAGCAAAATCCTAGACG	qRT-PCR
CAO-F	GATCCATACCCGATCGACAT	qRT-PCR
CAO-R	CGAGAGACATCCGGTAGAGC	qRT-PCR
DVR-F	CGAGCCCAGGTTCATCAAGGTGC	qRT-PCR
DVR-R	CCTCCCGATCTTGCCGAACTCC	qRT-PCR
OsLHCB2-F	TGTGTACACTTGTAGTAGCCAC	qRT-PCR
OsLHCB2-R	CACACACACACACAATTAAGGT	qRT-PCR
OsRBCS2-F	CATCTCAAGAAGTACTCGAGCA	qRT-PCR
OsRBCS2-R	GAACTTCTTGATGCCCTCAATC	qRT-PCR
OsPPI2-F	AAGGTTCTGCTAGTGTTAGTCC	qRT-PCR
OsPPI2-R	GAATCATCAAGGACAATCTGCC	qRT-PCR

Table S2 Accession numbers of RpoB, NdhB, NdhD, and homologous of OsPPR16 in 79 land plants.

Organism	OsPPR16	RpoB	NdhB	NdhD
Aegilops tauschii subsp. tauschii	XP_020185083.1	YP_008474290.1	YP_008474344.1	YP_008474353.1
Amborella trichopoda	XP_011624337.1	NP_904091.2	NP_904144.1	NP_904149.1
Ananas comosus	XP_020114486.1	YP_009116334.1	YP_009116387.1	YP_009116392.1
Arabidopsis lyrata subsp. lyrata	XP_002892635.1	YP_009357259.1	YP_009357327.1	YP_009357317.1
Arabidopsis thaliana	NP_172596.1	NP_051051.1	NP_051103.2	NP_051109.2
Asparagus officinalis	XP_020241333.1	YP_009370013.1	YP_009370065.1	YP_009370070.1
Brachypodium distachyon	XP_010228112.1	YP_002000478.1	YP_002000529.1	YP_002000535.1

Brassica napus	XP_022547046.1	YP_005089944.1	YP_005090013.1	YP_005090003.1
Brassica oleracea var. oleracea	XP_013604251.1	YP_009564567.1	YP_009564620.1	YP_009564626.1
Brassica rapa	XP_018509990.1	YP_009553759.1	YP_009553812.1	YP_009553818.1
Cajanus cajan	XP_020225218.1	YP_009309154.1	YP_009309198.1	YP_009309208.1
Camelina sativa	XP_019098944.1	YP_009231067.1	YP_009231119.1	YP_009231125.1
Camellia sinensis	XP_028089616.1	YP_007317240.1	YP_007317309.1	YP_007317299.1
Cannabis sativa	XP_030480596.1	YP_009123065.1	YP_009123116.1	YP_009123122.1
Capsella rubella	XP_006304494.1	YP_009161913.1	YP_009161965.1	YP_009161970.1
Capsicum annuum	XP_016560017.1	YP_006666022.1	YP_006666075.1	YP_006666080.1
Carica papaya	XP_021901338.1	YP_001671675.1	YP_001671727.1	YP_001671732.1
Chenopodium quinoa	XP_021745221.1	YP_009380122.1	YP_009380174.1	YP_009380180.1
Cicer arietinum	XP_004499782.1	YP_002149733.1	YP_002149775.1	YP_002149785.1
Citrus sinensis	XP_006494629.1	YP_740467.1	YP_740521.1	YP_740527.1
Coffea arabica	XP_027114312.1	YP_817474.1	YP_817527.1	YP_817532.1
Coffea canephora	CDP18169.1	YP_009251117.1	YP_009251170.1	YP_009251176.1
Cucumis sativus	XP_004147126.2	YP_247591.1	YP_247644.1	YP_247650.1
Cucurbita maxima	XP_022967032.1	YP_009447442.1	YP_009447495.1	YP_009447501.1
Cucurbita moschata	XP_022945178.1	YP_009447527.1	YP_009447597.1	YP_009447586.1
Durio zibethinus	XP_022765697.1	YP_009457853.1	YP_009457906.1	YP_009457911.1
Elaeis guineensis	XP_010940790.1	YP_006073096.1	YP_006073149.1	YP_006073154.1
Eucalyptus grandis	XP_010067671.1	YP_003933954.1	YP_003934003.1	YP_003934008.1
Eutrema salsugineum	XP_006393717.1	YP_009175667.1	YP_009175736.1	YP_009175726.1
Glycine max	XP_003526349.1	YP_538763.1	YP_538808.1	YP_538818.1

Glycine soja	XP_028238335.1	YP_008816245.1	YP_008816289.1	YP_008816299.1
Gossypium arboreum	XP_017643513.1	YP_005088991.1	YP_005089042.1	YP_005089052.1
Gossypium hirsutum	XP_016746578.1	YP_538927.1	YP_538980.1	YP_538990.1
Gossypium raimondii	XP_012449363.1	YP_005087685.1	YP_005087736.1	YP_005087746.1
Helianthus annuus	XP_021970029.1	YP_588104.1	YP_588162.1	YP_588173.1
Hevea brasiliensis	XP_021639377.1	YP_004327649.1	YP_004327721.1	YP_004327711.1
Ipomoea nil	XP_019175148.1	YP_009295096.1	YP_009295148.1	YP_009295157.1
Jatropha curcas	XP_012068740.1	YP_002720104.1	YP_002720157.1	YP_002720162.1
Juglans regia	XP_018849370.1	YP_009186160.1	YP_009186228.1	YP_009186218.1
Lactuca sativa	XP_023768602.1	YP_398316.1	YP_398373.1	YP_398378.2
Manihot esculenta	XP_021628108.1	YP_001718429.1	YP_001718481.1	YP_001718486.1
Medicago truncatula	XP_013458907.1	YP_001381728.1	YP_001381686.1	YP_001381674.1
Momordica charantia	XP_022139093.1	YP_009456053.1	YP_009456105.1	YP_009456112.1
Morus notabilis	XP_010091324.1	YP_009139671.1	YP_009139723.1	YP_009139728.1
Musa balbisiana	THU44351.1	YP_009180363.1	YP_009180416.1	YP_009180424.1
Nelumbo nucifera	XP_010243611.1	YP_009093942.1	YP_009093994.1	YP_009093999.1
Nicotiana attenuata	XP_019224183.1	YP_009429442.1	YP_009429494.1	YP_009429499.1
Nicotiana sylvestris	XP_009799583.1	YP_358664.1	YP_358727.1	YP_358737.1
Nicotiana tabacum	XP_016505961.1	NP_054488.1	NP_054547.1	NP_054557.2
Nicotiana tomentosiformis	XP_009631335.1	YP_398851.1	YP_398913.1	YP_398923.1
Oryza brachyantha	XP_006653155.1	YP_009266412.1	YP_009266465.1	YP_009266471.1
Oryza sativa Japonica Group	XP_015643851.1	NP_039373.1	NP_039432.1	NP_039444.1
Papaver somniferum	XP_026424340.1	YP_009234974.1	YP_009235027.1	YP_009235032.1

Phoenix dactylifera	XP_026656065.1	YP_003540923.1	YP_003540976.1	YP_003540983.1
Physcomitrella patens	XP_024389794.1	NP_904223.1	NP_904166.1	NP_904238.1
Populus euphratica	XP_011020490.1	YP_009054421.1	YP_009054473.1	YP_009054478.1
Populus trichocarpa	XP_002303960.2	YP_001109491.1	YP_001109548.1	YP_001109557.2
Prunus avium	XP_021832939.1	YP_009691926.1	YP_009691977.1	YP_009691982.1
Prunus mume	XP_008231609.1	YP_009020030.1	YP_009020083.1	YP_009020088.1
Prunus persica	XP_007220255.1	YP_004021657.1	YP_004021709.1	YP_004021714.1
Raphanus sativus	XP_018439963.1	YP_009046906.1	YP_009046959.1	YP_009046965.1
Ricinus communis	XP_015578720.1	YP_005090170.1	YP_005090222.1	YP_005090228.1
Selaginella moellendorffii	XP_024537389.1	YP_003097476.1	YP_003097473.1	YP_003097525.1
Sesamum indicum	XP_011101861.1	YP_004935658.1	YP_004935712.1	YP_004935717.1
Setaria italica	XP_012700613.1	YP_008815741.1	YP_008815794.1	YP_008815800.1
Setaria viridis	TKW19248.1	YP_009172130.1	YP_009172183.1	YP_009172190.1
Solanum lycopersicum	XP_004233812.1	YP_008563080.1	YP_008563134.1	YP_008563139.1
Solanum pennellii	XP_015063616.1	YP_009421349.1	YP_009421403.1	YP_009421408.1
Solanum tuberosum	XP_006347159.1	YP_635631.1	YP_635684.1	YP_635689.2
Sorghum bicolor	XP_021318540.1	YP_899397.1	YP_899451.1	YP_899457.1
Spinacia oleracea	XP_021838361.1	NP_054924.1	NP_054977.1	NP_054988.2
Tarenaya hassleriana	XP_010540194.1	YP_009356519.1	YP_009356571.1	YP_009356577.1
Theobroma cacao	XP_017984844.1	YP_004021309.1	YP_004021359.1	YP_004021369.1
Triticum aestivum	SPT16892.1	NP_114249.1	NP_114301.1	NP_114308.1
Vigna radiata var. radiata	XP_014499600.1	YP_003434344.1	YP_003434384.1	YP_003434389.1
Vigna unguiculata	XP_027939951.1	YP_006460343.1	YP_006460382.1	YP_006460388.1

Vitis vinifera	XP_01907316 3.1	YP_567068 .1	YP_567123.1	YP_567128.1
Zea mays	NP_00135656 9.1	NP_043015 .1	NP_043076.1	NP_043087.1
Ziziphus jujuba	XP_01587614 2.1	YP_009256 319.1	YP_0092563 72.1	YP_0092563 77.1

Table S3 Genotype and phenotype of T_0 transgenic plants.

Line	Genotype	Phenotpye
osppr16d-1	Biallelic mutant	Pale
osppr16d-2	Biallelic mutant	Pale
osppr16d-3	Biallelic mutant	Pale
osppr16d-4	Biallelic mutant	Pale
OsPPR16d-5	Wild-type	Green
osppr16d-6	Biallelic mutant	Pale
OsPPR16d-7	Heterozygous	Green
OsPPR16d-8	Heterozygous	Green
OsPPR16d-9	Wild-type	Green
osppr16d-10	Biallelic mutant	Pale
OsPPR16d-11	Heterozygous	Green
OsPPR16d-12	Wild-type	Green
OsPPR16d-13	Heterozygous	Green
OsPPR16d-14	Heterozygous	Green
osppr16d-15	Biallelic mutant	Pale
osppr16d-16	Biallelic mutant	Pale
OsPPR16d-17	Wild-type	Green
OsPPR16d-18	Heterozygous	Green
OsPPR16d-19	Heterozygous	Green
OsPPR16d-20	Wild-type	Green
osppr16d-21	Biallelic mutant	Pale
OsPPR16d-22	Heterozygous	Green
OsPPR16d-23	Heterozygous	Green
osppr16d-24	Biallelic mutant	Pale
OsPPR16d-25	Heterozygous	Green
OsPPR16d-26	Wild-type	Green
OsPPR16d-27	Heterozygous	Green
OsPPR16d-28	Wild-type	Green
OsPPR16d-29	Heterozygous	Green
OsPPR16d-30	Heterozygous	Green

OsPPR16d-31	Heterozygous	Green
OsPPR16d-32	Heterozygous	Green
OsPPR16d-33	Wild-type	Green
OsPPR16d-34	Heterozygous	Green
OsPPR16d-35	Heterozygous	Green
osppr16d-36	Homozygous	Pale
OsPPR16s-1	Wild-type	Green
OsPPR16s-2	Heterozygous	Green
OsPPR16s-3	Heterozygous	Green
osppr16s-4	Biallelic mutant	Pale
OsPPR16s-5	Wild-type	Green
OsPPR16s-6	Heterozygous	Green
osppr16s-7	Biallelic mutant	Pale
OsPPR16s-8	Heterozygous	Green
OsPPR16s-9	Heterozygous	Green
OsPPR16s-10	Wild-type	Green
osppr16s-11	Biallelic mutant	Pale
OsPPR16s-12	Wild-type	Green
OsPPR16s-13	Heterozygous	Green
osppr16s-14	Biallelic mutant	Pale
OsPPR16s-15	Heterozygous	Green
OsPPR16s-16	Heterozygous	Green
osppr16s-17	Homozygous	Pale
OsPPR16s-18	Wild-type	Green
OsPPR16s-19	Heterozygous	Green
OsPPR16s-20	Heterozygous	Green
OsPPR16s-21	Wild-type	Green
OsPPR16s-22	Heterozygous	Green
osppr16s-23	Biallelic mutant	Pale
OsPPR16s-24	Heterozygous	Green
OsPPR16s-25	Heterozygous	Green
OsPPR16s-26	Wild-type	Green
osppr16s-27	Biallelic mutant	Pale
OsPPR16s-28	Heterozygous	Green
OsPPR16s-29	Heterozygous	Green
OsPPR16s-30	Heterozygous	Green
osppr16s-31	Biallelic mutant	Pale
OsPPR16s-32	Heterozygous	Green
osppr16s-33	Homozygous	Pale

osppr16s-34	Biallelic mutant	Pale

Methods S1 Complementation of the *osppr16* mutant.

For mutant complementation, an 8300-bp WT genomic DNA fragment comprising the entire *OsPPR16* coding region, 4731 bp of the 5' upstream region and 1364 bp of 3' downstream sequence was cloned into the pCAMBIA2300 vector (CAMBIA, Canberra, Australia) in two steps. In the first step, a 5533 bp DNA fragment (3' portion of the 8300-bp fragment) was amplified with primers CO16A-F and CO16A-R, digested with *Sac* I and *Sal* I, and ligated into pCAMBIA2300 to generate pCAMBIA2300-OsPPR16A. In the second step, a 2767 bp DNA fragment (5' portion of the 8300-bp fragment) was amplified with primers CO16B-F and CO16B-R and subcloned into the pCAMBIA2300-OsPPR16A vector (digested with *Sac* I) by using the ClonExpress II One Step Cloning Kit (Vazyme, Nanjing, China). The pCAMBIA2300-OsPPR16 vector was transformed into callus tissue induced from mature seeds of line *osppr16d-36-6*, which was free of the CRISPR/Cas9 construct. The sequences of all primers are listed in Table S1.

Reference

Liere K, Börner T 2007. Transcription and transcriptional regulation in plastids. *Cell and molecular biology of plastids*: Springer, 121-174.
